# Sustainable utilization of the vegetable oil manufacturing waste product in the formulation of eco-friendly emulsifiable cutting fluids

**DOI:** 10.1038/s41598-023-46768-8

**Published:** 2023-12-04

**Authors:** Toka Hassan, Eman M. Kandeel, M. S. Taher, Entsar E. Badr, A. S. El-Tabei

**Affiliations:** 1 Chemistry Department, Faculty of Science, Al-Azhar University (for Girls), Cairo, 11754 Egypt; 2 Egyptian Petroleum Research Institute (EPRI), Nasr City, Cairo, 11727 Egypt

**Keywords:** Environmental chemistry, Green chemistry, Surface chemistry, Environmental sciences, Materials science

## Abstract

The conventional Metal cutting fluids (MCFs) used are mineral-based petroleum oils that perform well but are toxic and difficult to dispose of; therefore, these are hazardous to human health as well as the environment. This issue can be solved by using natural vegetable oil-based MCF, which are readily available, environment and human-friendly, and renewable. Therefore, we synthesized various types of emulsifiers (anionic, and nonionic with different ethylene oxide units as well as mono and gemini cationic surfactants as corrosion inhibitors and biocides) based on recycled vegetable oil (RO) from spent bleaching earth (SBE), and elucidated their chemical structures by different spectroscopic techniques. The individually synthesized emulsifiers (anionic, and nonionic with different ethylene oxide units) at different ratios (8–15 by wt.%) and mixed emulsifiers (anionic/nonionic, nonionic/nonionic with different degrees of ethylene oxide) at different ratios (8–12 by wt.%) were utilized as additives in the preparation of different vegetable residual oil-based MCF formulations. The mixed emulsifiers at different ratios of nonionic/nonionic with hydrophilic-lipophilic balance (HLB) value 10 (Formulas I, II, III, and IV), and anionic/nonionic (Formula V, and VI) exhibited stable emulsions compared to individual emulsifiers. Formulas (I and VI) displayed good protection effectiveness in corrosion tests. Formula VI had better wettability (25.22 on CS, 23.68 on Al, and 22.28 on WC) and a smaller particle size (63.97 nm). Tribological properties of Formula VI were also performed. The results exhibit that Formula VI is consistent with the commercial sample. As a result, this study contributed to the resolution of one of the industry's problems

## Introduction

In the edible oil refining industry, bleaching earth (BE) is utilized to eliminate color, residual gums, phospholipids, and metals from the oil^1,2^. Also, it absorbs varying amounts of edible oils according to the type of oil and the technologies used. Spent bleaching earth (SBE) is a waste oil refinery having a high proportion of vegetable oil (~ 20–40%)^3–5^. Significant volumes of SBE are discarded in landfills, which cause fire and pollution risks owing to the large oil content in the earth which leads to climate changes^6,7^. SBE oil would have been wasted if it had not been retrieved which would have caused oil depletion in the long term. Thus, oil preserved in SBE can be recycled and reused in industrial applications as raw materials in order to reduce costs in the oil refining industry^8–14^. However, The recycled vegetable oil would have high free fatty acids and peroxide value, and would not be suitable for food application^12^. Recently, the application of vegetable oil-based metal cutting fluids (MCFs) has been proposed. MCFs are an essential aspect of the modern industrial system. During the metal machining process; a high temperature at the cutting zone is produced by the friction between the cutting tool-work piece and cutting tool-chip interfaces. This heat causes a shorter lifetime of the tool, a higher roughness of the surface, and decreases the dimensional sensitivity of the work material. This finding is more significant when machining difficult-to-cut materials owing to higher heat occurrence^15^. One of the methods to reduce the heat generated at the tool-workpiece interface is the use of MCFs in machining operations^15^. As a result, the current demand for MCFs is increasing^16^ and the development of new MCFs with superior performance based on waste products is required^17^. MCFs are used during the machining operations to provide lubrication and cooling effects between the cutting tool-work piece and the cutting tool-chip. Hence, the heat will be carried away by the lubricant during the cutting process^17–21^. Also, they facilitate chip flushing away from the machining zone and prevent adhesion of the workpiece to the cutting tool.

High temperature is the primary cause of tool wear in dry cutting, resulting in tool failure. As a result, the presence of coolant is critical in machining. Cooling techniques used in machining include conventional cooling, cryogenic cooling, minimum quantity lubrication (MQL), nano-fluid machining, and air cooling. MQL; most typically used in metal cutting operations: delivers a combined action of coolant and lubricant, which reduces setup and fluid costs. MQL fluid evaporates at the cutting zone, eliminating the need for cutting fluid maintenance, circulation, and disposal^22^. The machining parameters improved utilizing minimum quantity lubrication are cutting temperature, surface roughness, tool wear, and cutting forces achieving good machinability of metal^23^.

The key ingredient of industrial metal cutting fluids is mineral oil and other additives with distinctive properties. Mineral oil-based MCFs are non-biodegradable and have a negative impact on the environment. Furthermore, these MCFs pose numerous health risks^24,25^. Toxic and corrosive slurries and fluids are employed in cutting and polishing fluids. This induces pollution in the environment. To solve this challenge, novel green fluids are proposed^26–31^. Using the novel green fluids, high-performance surfaces are manufactured for use in semiconductor, optoelectronics, and aerospace industries^32^. These novel green fluids are a great contribution to conventional manufacturing, and the most important is reducing the pollution to the environment effectively^33^.

Therefore, the formulation of metal cutting fluid dependent on vegetable oils as a renewable source is regarded as one of the industry's unique solutions^20,24^. It would be very appealing to replace petroleum-based MCFs with bio-based MCFs (vegetable oil-based MCFs), which are environmentally friendly, renewable, less toxic, and highly biodegradable. Consequently, in addition to having superior coolant and lubricant properties, high-quality MCFs should be safe to discard after use^34–36^.

As a result, this research focuses on optimizing the benefits of recycled vegetable oil as a base ingredient for the preparation of various types of surface active agents. The synthesized surfactants are evaluated as emulsifiers, corrosion inhibitors, and biocides and they’re applied in designing various metal-cutting fluid formulations. Thus, the synthesized surfactants fulfill all the requirements of cutting fluid formulations. Additionally, recycled vegetable oil is used as a vegetable oil base in metal-cutting fluid formulations.

In this study, residual vegetable oil recovery was extracted from SBE and chemically modified into multifunctional surfactant compounds which were utilized as additives in the preparation of different vegetable oil-based metal-cutting fluid formulations. In addition, the role of individual and mixed emulsifiers at different ratios in the stabilization of the formulations was studied. These formulations were evaluated using oil stability, emulsion stability; corrosion inhibition, wettability, particle size, and wear resistance tests and then compared these data with commercial sample results.

## Experimental methods

### Spent bleaching earth

Spent bleaching earth (SBE) was provided by Tanta Oil and Soap Company. Soybean oil accounts for 75% of domestic oil production. Consequently, the SBE sample used is taken from refining soybean oil.

#### Extraction of oil from spent bleaching earth

**Figure 1:**
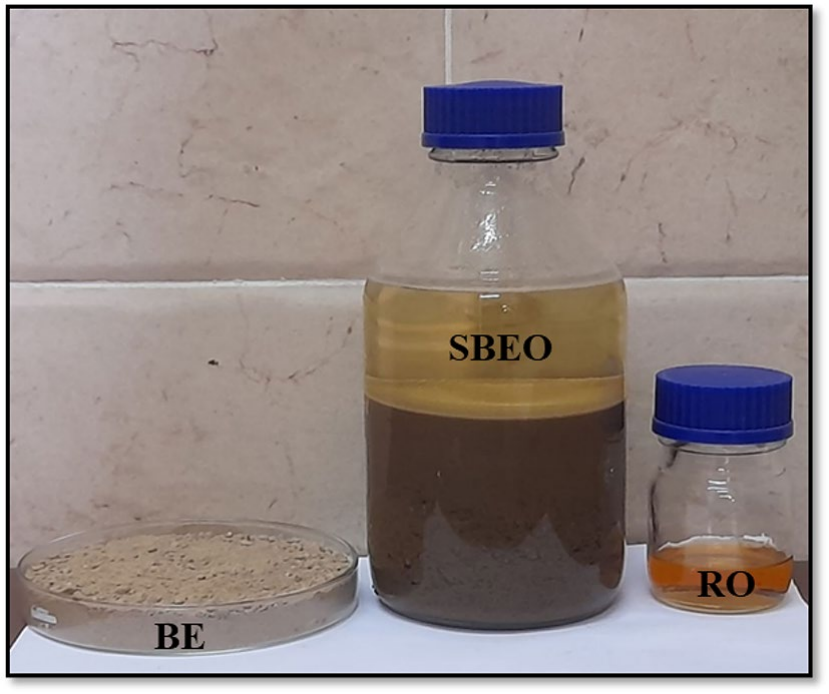
Extraction process of recycled vegetable oil from spent bleaching earth.

Cold extraction with n-hexane solvent was used to extract the oil from SBE. One liter of n-hexane was put into a 2L bottle holding 0.5 kg of fresh SBE. The bottle was tightly closed and set aside for 72 h at room temperature. Afterward, the resulting solution was decanted and filtered. The solution (oil + n-hexane) was then concentrated by distillation utilizing a vacuum pump to collect the recycled vegetable oil. From the 0.5 kg SBE, 55 g of oil was collected, producing an oil yield of 11%. The solvent that was recovered might be used again^14^ as shown in Fig. 1.

### Analysis

Physicochemical properties of the recycled vegetable oil such as density, color, viscosity, saponification value, free fatty acid, total acid value, iodine value, peroxide value, flash point, molecular weight, and fatty acid composition were reported in Table 1.

**Table 1:** Physicochemical properties of recycled vegetable oil.

Property	Refined soybean oil	Recycled vegetable oil from SBE (soybean oil)	Oil extracted from SBE (soybean oil)^12^*
Density (kg/m^3^)	0.912	0.889	0.88
Color	Pale yellow	Yellow	Dark brown
Viscosity (cP at 25 °C)	39.5	65.5	84–96
Saponification value (mg/g)	198.95	209.3	185
Free fatty acid (FFA) %	0.32	53.67	24.1
Total acid value (mg/g)	0.64	89.9	87.6
Iodine value (g/100g)	122.6	64.7	–
Peroxide value (meg O_2_/kg)	50.76	76.44	–
Flash point	262.2	289.7	–
Molecular weight (g/mol)	829.61	858.45	–
Fatty acid composition (%)			
Palmitic acid (C16:0)	10.44	34.79	57.9
Stearic acid (C18:0)	4.67	15.69	22.6
Oleic acid (C18:1)	24.79	41.45	19.5
Linoleic acid (C18:2)	57.8	8.08	0
Linolenic acid (C18:3)	2.3	0	0
Reference	This work	This work	^12^

#### Fatty acid composition

The recycled vegetable oil's fatty acid composition was identified via a modified method^37^ with HP 6890 plus gas chromatography (Hewlett Packard, USA).

### Synthesis of anionic emulsifier based on recycled vegetable oil

**Figure 2:**
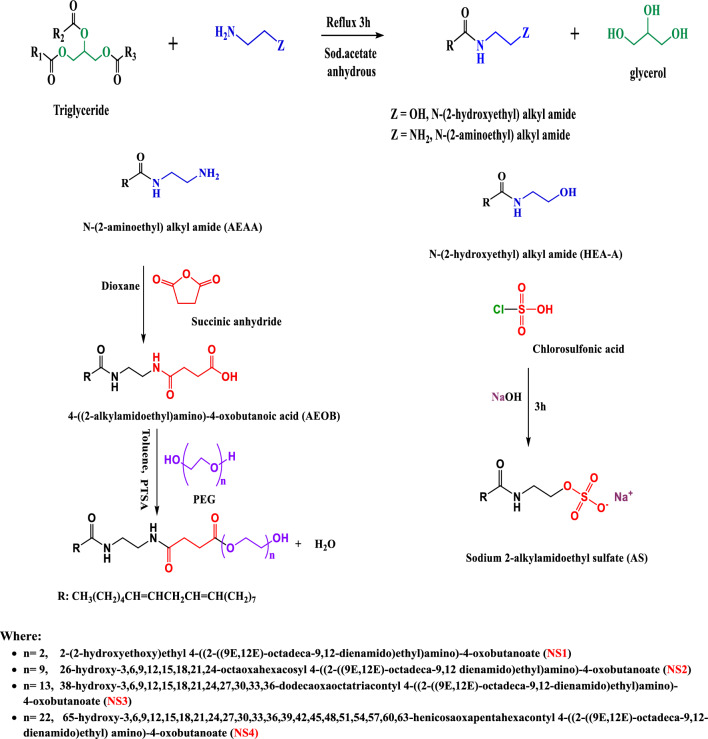
Synthesis of anionic (AS) and nonionic emulsifiers (NSs).

The synthesis of anionic emulsifier was illustrated in Fig. 2. The anionic emulsifier was synthesized in two steps as follows:

#### Amidation of recycled vegetable oil

In a three-neck round-bottom flask, 0.1 mol RO, monoethanolamine or ethylenediamine (0.31 mol), and 0.2 wt.% anhydrous sodium acetate was agitated for 3 h at 160 °C. The reaction product was dissolved in dichloromethane, washed three times with distilled water, and dried over anhydrous Na_2_SO_4_ to obtain yellow pasty N-alkyl ethanolamine N-(2-hydroxyethyl) alkyl amide (HEA-A) or N-(2-aminoethyl) alkyl amide (AEAA)^38^.

#### Synthesis of sodium 2-alkylamidoethyl sulfate (AS) via sulfation of N-(2-hydroxyethyl) alkyl amide (HEA-A)

N-(2-hydroxyethyl) alkyl amide (10.02 g, 0.031 mol) was dissolved in 20 ml chloroform at 5 °C, chlorosulfonic acid (3.96 g, 0.034 mol) was added dropwise slowly for 6 min, swirling the mixture magnetically. At the same temperature, stirring was continued for an additional three hours. n-Butanol was used to dilute the contents, and 18 N aqueous sodium hydroxide solution was used to neutralize them. The mixture of water and n-butanol was separated, and the residual material was dried at 80 °C under reduced pressure to obtain a pale brown waxy product^39,40^.

### Synthesis of nonionic emulsifiers derived from recycled vegetable oil

The synthesis of nonionic emulsifiers was illustrated in Fig. 2. The nonionic emulsifiers were synthesized as follows:

#### Synthesis of 4-((2-alkylamidoethyl)amino)-4-oxobutanoic acid (AEOB)

N-(2-aminoethyl) alkyl amide AEAA (35.48 g; 0.11 mol), succinic anhydride (10.00 g; 0.10 mol) were dissolved in 1,4-dioxane and agitated for 15 h at 80 °C. The solvent was removed under reduced pressure and the subsequent product was washed with diethyl ether, filtered off as a pale yellow solid with m.p. 105–110 °C^41^.

#### Esterification of 4-((2-alkylamidoethyl)amino)-4-oxobutanoic acid (NSs)

In 100 mL round bottom flask was charged with 4-((2-alkylamidoethyl)amino)-4-oxobutanoic acid (8.48 g, 20.07 mmol), polyethylene glycol with different molecular weight (Mn = 106, 400, 600, and 1000) (33.26 mmol), and p-toluene sulfonic acid (1.18 g, 6.88 mmol). Toluene was added as a solvent, and the mixture was refluxed for 8 h using a Dean-Stark apparatus. After cooling to ambient temperature, the mixture was treated with dichloromethane, then three times washed with water, dried over anhydrous Na_2_SO_4_, and concentrated under reduced pressure. The four nonionic emulsifiers (NS1-4) were obtained as viscous liquids^42^.

### Synthesis of mono and gemini cationic surfactants based on recycled vegetable oil

The synthesis of mono and gemini cationic surfactants was illustrated in Fig. (S1). The mono and gemini cationic surfactants were synthesized in two steps as follows:

#### Synthesis of N-(2-alkylamidoethyl)-2-chloroacetamide (AECA)

In a 500 ml round bottom flask, N-(2-aminoethyl) alkyl amide **AEAA** (29.03 g, 90 mmol) was dissolved in dichloromethane. The organic solution was mixed with 14.31 g of 135 mmol of Na_2_CO_3_ aqueous solution. The resulting two-phase mixture was cooled to 5 °C. A solution of chloroacetyl chloride (15.25 g, 135 mmol) in dichloromethane was added dropwise to the cooled solution for around 40 min while maintaining at 5 °C temperature. Then, the mixture was stirred for two hours at room temperature. The washing of the aqueous solution was done with dichloromethane three times, all of the organic solutions were added, then rinsed with water and dried over anhydrous Na_2_SO_4_ to get a brown waxy product^43^.

#### Synthesis of 2-((2-alkylamidoethyl)amino)-N,N,N-triethyl-2- oxoethan-1-aminium chloride (MCS)

In a 250 ml round bottom flask, N-(2-alkylamidoethyl)-2-chloroacetamide (AECA) (26 mmol, 10.37 g), and a solution of triethylamine (26 mmol, 2.63 g) in ethyl acetate was added and stirring under reflux 24 h. Under reduced pressure, the solvent was eliminated, and the crude product underwent three washes with diethyl ether to produce a white precipitate; m.p: 91–96 °C. The product was dried and stored in desiccators to prevent moisture absorption^44^.

#### Synthesis of N,N′-bis(2-((2-alkylamidoethyl)amino)-2-oxoethyl)- N,N,N′,N′-tetramethylethane-1,2-diaminium chloride (GCS)

The mixture of N,N,N′,N′-Tetramethylethane-1,2-diamine and N-(2-alkylamidoethyl)-2-chloroacetamide (AECA) in a molar ratio of 1:2 in ethyl acetate was refluxed for 48 h. A white precipitate was formed during the reaction and increased after completed the reaction time. The resulting product was washed with n-hexane and recrystallized in absolute ethanol to get an off-white precipitate with m.p. 98–102 °C. The product was dried and stored in desiccators to prevent moisture absorption^45^.

### Structural confirmation of the synthesized compounds

The chemical structures of all synthesized compounds were confirmed by IR spectra (KBr, υ cm) and were recorded on the CARY 630 FT-IR spectrometer (Agilent, Santa Clara, CA, USA). Also, NMR spectra were measured in (DMSO-d6) on a Bruker Avance (III) NMR spectrometer at 400 MHz; ^1^H & ^13^C-NMR at 400, and 100 MHz respectively: (Bruker, Switzerland). Finally, the mass spectra were attained through a GC Ms-QP 1000 EX mass spectrometer (Shimadzu, Kyoto, Japan) at 70 eV.

### Antimicrobial activity of mono and gemini cationic surfactants

The antimicrobial potential of mono and gemini cationic surfactants has been assessed according to the agar plate diffusion method^46^.

### Formulation of different cutting fluid emulsions or emulsifiable soluble oils using individual emulsifiers and mixture emulsifiers

The formulations of cutting fluid emulsions or emulsifiable soluble oil blends were prepared as follows^47^:

A cutting oil package has been formulated from different ratios of vegetable base oil (recycled vegetable oil) (78–89 by wt.%), individually synthesized emulsifiers (anionic, and nonionic with different ethylene oxide units) (8–15 by wt.%) or mixed synthesized emulsifiers (anionic/nonionic, nonionic/nonionic with different degrees of ethylene oxide) (8–12 by wt.%), oiliness or lubricant (oleic acid) (1–4 by wt.%), anticorrosion and biocide (GCS) (1–3 by wt.%) and coupling agent (diethylene glycol) (1–3 by wt.%). The mixture was blended with each other by magnetic stirring. Each ingredient in this mixture was tested individually at various concentrations to find the optimal concentration at which oil blend stability occurs. The performance of prepared cutting oil formulas was assessed based on cutting oil package stability and emulsion stability.

### Evaluation methods of prepared metal cutting fluid emulsion formulas

#### Oil stability (screening test)

In accordance with the standard procedure (IP311), put the test oil package into specimen bottles. All of the bottles were sealed, and placed in the oven at 50 °C as well as 0 °C for the duration of at least 15 h and at most 20 h. After this period, remove the bottles and immediately inspect the oil for any indications of turbidity, separation, or gelling; the blend that produces stable oil (no gel formation, no separation) was chosen for further examination.

#### Emulsion stability test

In accordance with the standard procedure (IP 263), all blend samples were examined for emulsion stability in the following way: Before testing, bring the oil package and water to a temperature of 25 °C. Utilizing the magnetic stirrer, stir the required amount of water in a 100 ml conical flask at a speed enough to produce a vortex just deep enough to reach the flask's bottom. Add the required amount of oil quickly and stir continuously for 2 min. Pour a sufficient amount of the emulsion into the graduated-neck flask. This flask was left for 24 h to assess emulsion stability (separation, gelation, or complete stability). Only emulsion-stable metal-cutting fluids (0.1 ml of cream or oil layer separation after 24 h) were chosen for further testing.

#### Corrosion test

In accordance with the standard procedure (ASTM D4627) for evaluating the anticorrosion test of metal cutting fluid emulsions, the cast iron chips were cleaned with benzene prior to use. Weigh 4.0 ± 0.1 g of cast iron chips and place them in a petri dish on filter paper. Put 5.0 ml of emulsions on cast iron chips. Use the stirring rod to ensure that all chips are submerged, all air bubbles have been released, and the chips are distributed evenly. The dish is covered and left to stand for 24 h. The amount of rust stain on the filter paper indicates the fluid's ability to control corrosion.

#### Surface tension measurements

The surface tension was assessed using the pendant drop technique utilizing Theta optical tensiometer, Biolin Scientific Company, Finland. The surface tension of various formulas was measured at 25 °C (298 K). To ensure the measurement's accuracy, water was examined.

#### Wettability test (contact angle)

The sessile drop method was used to measure the contact angle with a Theta Optical Tensiometer, Biolin Scientific Company, Finland. The computer automatically fits the droplet profile and computes the contact angle. Polished carbon steel (CS), aluminum (Al) 6061, and tungsten carbide (WC) surfaces were used to mimic the carbon steel, aluminum-based workpiece, and the tool material respectively, and the contact angle was measured on each. Between tests, the sample surfaces were cleaned with ethanol and dried.

#### Droplet size

The droplet size distribution of the prepared formulas was measured by using dynamic light scattering (Malvern Zetasizer ZS, Worcestershire, UK) at 25 °C.

#### Photographic image

A ZEISS Axiolab 5 digital laboratory optical polarizing microscope equipped with a Leica MC190 HD microscope camera was utilized in order to perform photographic microscopy tests for FI, FVI and commercial sample at 25 °C (298 K). On a glass slide, an emulsion droplet was spread out and exanimated.

#### Tribology studies

A tribology measuring cell connected to Physica MCR-502 controlled-stress rheometers was used to conduct tribological tests. A ball-on-pyramid principle governs the setting of the tribology accessory A 6.35 mm diameter steel ball (1.4401 grade 100) rotating on three steel plates (1.4301) at a 45° angle is used in the cell. The evolution of the friction factor with sliding velocities was studied for the normal load (10 N) and 900 s at 25 °C.

#### Cytotoxicity test

In vitro, cytotoxicity of two MCF samples (commercial sample and FVI) was assessed by MTT assay. Samples were investigated for their cytotoxicity effect against human keratinocyte line (HaCaT cell line). For each sample, different concentrations ug/ml (312.5, 625, 1250, 2500, 5000, 10,000) were prepared. Growth medium was decanted from 96 well micro titer plates after confluent sheet of Vero-e6 cell was formed; cell monolayer was washed twice with wash media. Double-fold dilutions of tested sample were made using Dulbecco’s Modified Eagle’s Medium (DMEM), where 0.1 ml of each dilution was tested in different wells leaving 3 wells as control, receiving only maintenance medium. Microplates were incubated at 37 °C and examined for 48 h. Cells were checked for any physical signs of toxicity. MTT solution was prepared (5 mg/ml in PBS) (BIO BASIC CANADA INC), and then 20ul MTT solution were added to each well, and shaked at 150 rpm for five minutes, to thoroughly mix the MTT into the media. Incubation for 4 h (37C, 5% CO_2_) was maintained to allow the MTT to be metabolized. The media were then dumped off and MTT metabolic product was re-suspend formazan in 200ul Dimethyl sulfoxide (DMSO). Plates were placed on a shaking table (150 rpm for 5 min) to thoroughly mix the formazan into the solvent. Optical Density (O.D.) at 560 nm was recorded and subtracts background at 620 nm. The maximum non-toxic concentration [MNTC] of each extract was determined and was used for further biological studies^48^.

## Results and discussion

### Physiochemical analysis of the recycled vegetable oil sample

Recycled vegetable oil from SBE using n-hexane was 11%, which was 17–19% lower than the amounts reported in the other studies^12,49^. When methanol, ethanol, or polar compounds were used as the solvent in the extraction step, a higher percentage could be obtained; however, the extracted oil contained pigments, suspended solids, polar and phosphorous compounds, making separation of the components difficult. Furthermore, the oil production is affected by the BE form and the feedstock utilized in the refinery according to its specific surface area. The composition of the feedstock determines the extraction yield, which is related to the melting point of the fatty acids. A lower melting point of feedstock makes oil extraction easier than a greater melting point of feedstock^50^. As a result, it is clear why our yield is low; the oil has approximately equal percentages of saturated and unsaturated fatty acids (UFAs).

Table 1 summarizes the chemical composition and characteristics of recycled vegetable oil from SBE as reported by various studies for comparison^14,51,52^. The oil was yellow in color, clear, and free of any suspended particles. While RO had a density comparable to refined soybean oil, its viscosity was 1.66 times higher, owing mostly to its increased amount of saturated fatty acids. The RO exhibited a saponification value (SV) of 209.30, surpassing that of the refined oil (198.95 mg of KOH/g of oil). Additionally, the RO had a significantly higher free fatty acid (FFA) content of 53.67%, in stark contrast to the refined soybean oil or recycled vegetable oil obtained from SBE as reported in the earlier studies^12,49^. The refined oil had an iodine value of 122.6 g/100 g, indicating a rather high degree of unsaturation^3^. In comparison to refined soybean oil, RO had the lowest iodine value of 64.7 g/100 g, owing to its higher percentage of saturated fatty acids. Additionally, the RO had a high peroxide value, indicating the existence of considerable amounts of hydro-peroxides generated via oil oxidation during the bleaching and storage operations^53^. It is also worth noting that RO oxidizes faster than refined oil, which can be related to FFAs' higher oxidation sensitivity relative to triglycerides^53,54^. Finally, the RO had a greater flash point, which contributed to a lower risk of smoke and fire dangers, hence improving safety in our specific application^55^.

The RO contained a notable proportion of saturated fatty acids, specifically palmitic acid (34.79%) and stearic acid (15.69%). In contrast, only 15% of the fatty acids in refined soybean oil were saturated. On the other hand, the RO exhibited significant amounts of unsaturated fatty acids, such as oleic acid (41.45%) and linoleic acid (8.08%), while refined soybean oil primarily consisted of unsaturated fatty acids (84.89%). The RO displayed a lower content of unsaturated fatty acids, particularly linoleic and linolenic acids, compared to refined soybean oil. This disparity can be attributed to the harsh conditions to which the SBE recycled vegetable oil was exposed, including light, oxygen, and high temperature, which accelerated the oxidation reactions of unsaturated fatty acids^3^.

### Chemical structure confirmation of synthesized anionic emulsifier based on recycled vegetable oil

The synthesis of an anionic emulsifier can be achieved in two steps. Firstly, recovered oil was subjected to amidation with monoethanolamine, using anhydrous sodium acetate as a catalyst to form N-(2-hydroxyethyl)alkyl amide (HEAA)^38^. Subsequently, the HEAA was sulfated with chlorosulfonic acid, to obtain Sodium 2-alkylamidoethyl sulfate (AS)^39,40^. The chemical reaction was illustrated in Fig. 2.

#### Chemical structure of N-(2-hydroxyethyl) alkyl amide (HEA-A)

FT-IR (KBr, *ν*_max_ cm^−1^) (Fig. S2): 3472.99(*ν*_OH_ stretch), 3296.19 (*ν*_NH_ stretch), 2919.07, 2850.18(*ν*_C–H_ asym. and sym. stretch), 1642.49 (*ν*_C=O_, amide).

^1^H-NMR spectrum (400 MHz, CDCl_3_, δ ppm): of **HEAA** (Fig. S3) demonstrated various peak at 0.80 (3H, C**H**_**3**_); 1.2 (14H, (C**H**_**2**_)_**7**_); 1.40 (2H, C**H**_**2**_CH_2_CO); 1.98 (2H, CH = CHC**H**_**2**_); 2.13 (2H, C**H**_2_C = O); 2.69 (2H, CH = CHC**H**_2_CH = CH) 3.32 (2H, C**H**_**2**_CH_2_OH); 3.57 (2H, NHCH_2_C**H**_**2**_); 4.40 (1H, O**H**); 5.26 (4H, C**H** = C**H**-CH_2_C**H** = C**H**) 6.9 (1H, O = C-N**H**-CH_2_).

#### Chemical structure of sodium 2-alkylamidoethyl sulfate (AS)

**Figure 3:**
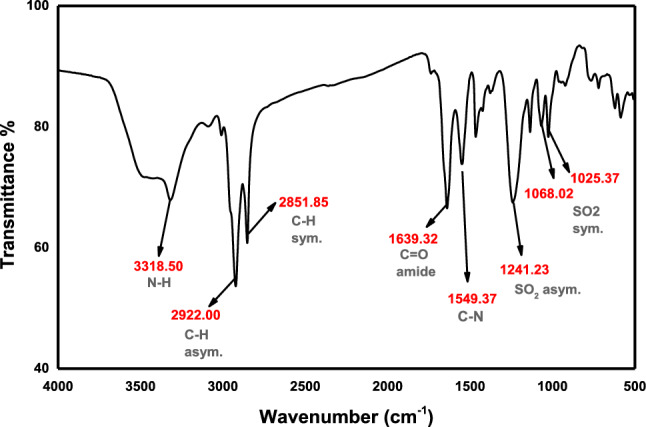
IR spectrum of sodium 2-alkylamidoethyl sulfate (AS).

FT-IR (KBr, *ν*_max_ cm^−1^) (Fig. 3): 3318.50 (*ν*_NH_ stretch), 2922.00, 2851.85(*ν*_C–H_ asym. and sym. stretch), 1639.32 (*ν*_C=O_, amide), 1549.37 (*ν*_C-N_ stretch), 1241.23, 1068.02, 1025.37 (*ν*_S=O_ asym. and sym. stretch.).

**Figure 4:**
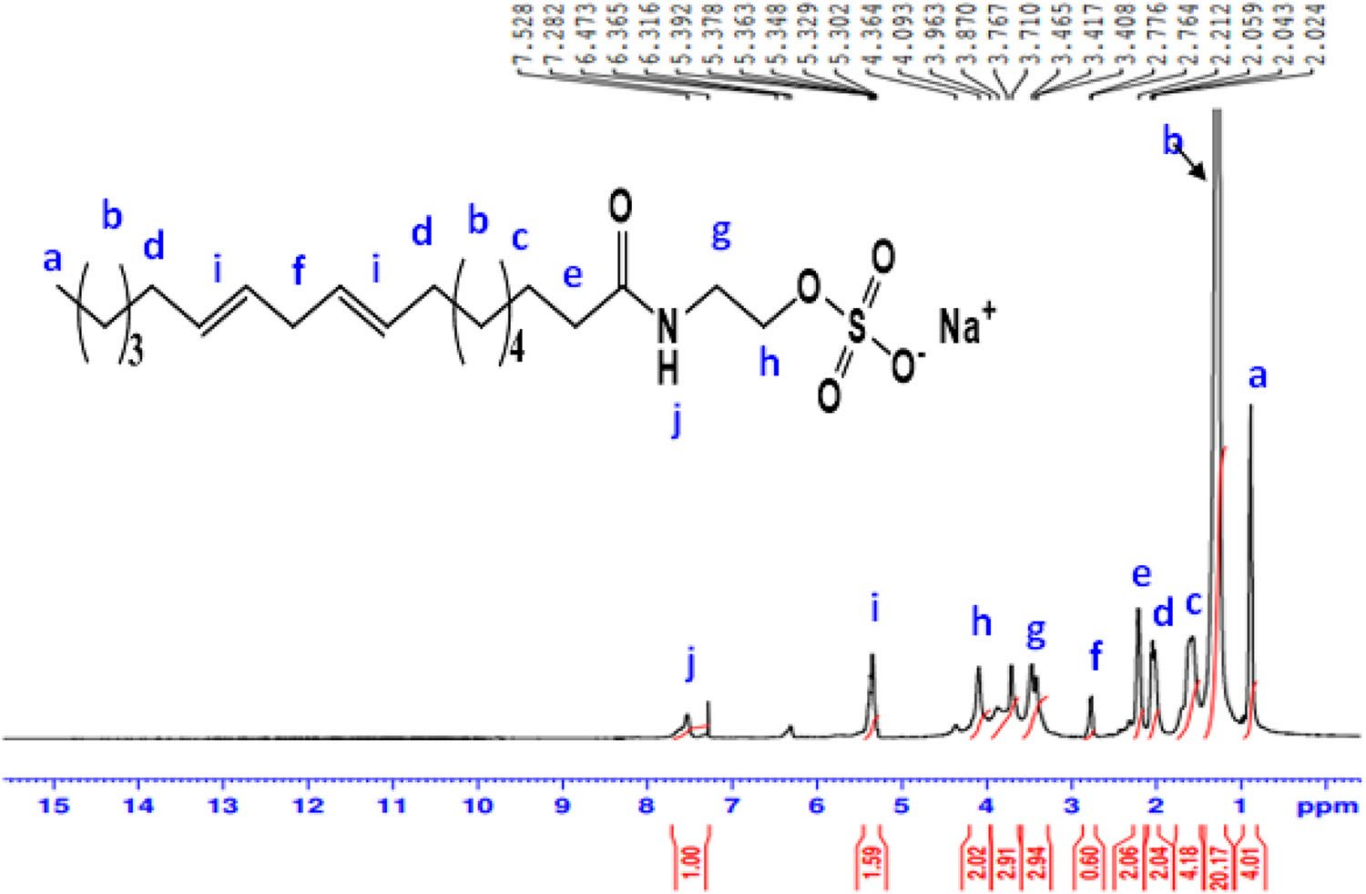
^1^H-NMR spectrum of Sodium 2-alkylamidoethyl sulfate (AS).

^1^H-NMR spectrum (400 MHz, CDCl_3_, δ ppm): of **AS** (Fig. 4) demonstrated various peak at 0.89 (3H, C**H**_**3**_); 1.3 (14H, (C**H**_**2**_)_**7**_); 1.5 (2H, C**H**_**2**_CH_2_CO); 1.97 (4H, C**H**_2_CH = CH); 2.21 (2H, C**H**_2_C = O); 2.77 (2H, CH = CHC**H**_2_CH = CH); 3.41 (2H, C**H**_**2**_CH_2_OSO_3_Na); disappearance of proton of OH and increase chemical shift of C**H**_**2**_ at 4.09 (2H, C**H**_**2**_OSO_3_Na); 5.37 (4H, C**H** = C**H**-CH_2_C**H** = C**H**); 7.52 (1H, O = C-N**H**-CH_2_).

### Chemical structure confirmation of synthesized nonionic emulsifiers based on recycled vegetable oil

The synthesis of nonionic emulsifiers can be accomplished in three steps. Firstly, the recovered oil was subjected to amidation with ethylenediamine using anhydrous sodium acetate as a catalyst to form N-(2-aminoethyl)alkyl amide (AEAA). Secondly, the reaction between (AEAA) and succinic anhydride produces 4-((2-alkylamidoethyl)amino)-4-oxobutanoic acid (AEOB)^41^. In the last step, nonionic emulsifiers (NS1-4) are obtained by reacting AEOB with polyethylene glycol of various molecular weights (106, 200, 400, 600, and 1000) in the presence of p-toluenesulfonic acid (PTSA) as a catalyst^42^. The chemical reaction was illustrated in Fig. 2.

#### Chemical structure of N-(2-aminoethyl) alkyl amide (AEAA)

FT-IR (KBr, *ν*_max_ cm^−1^) (Fig. S4): 3304.24 (*ν*_NH_ stretch), 2921.04, 2850.97(*ν*_C–H_ asym. and sym. stretch), 1640.87 (*ν*_C=O_, amide).

**Figure 5:**
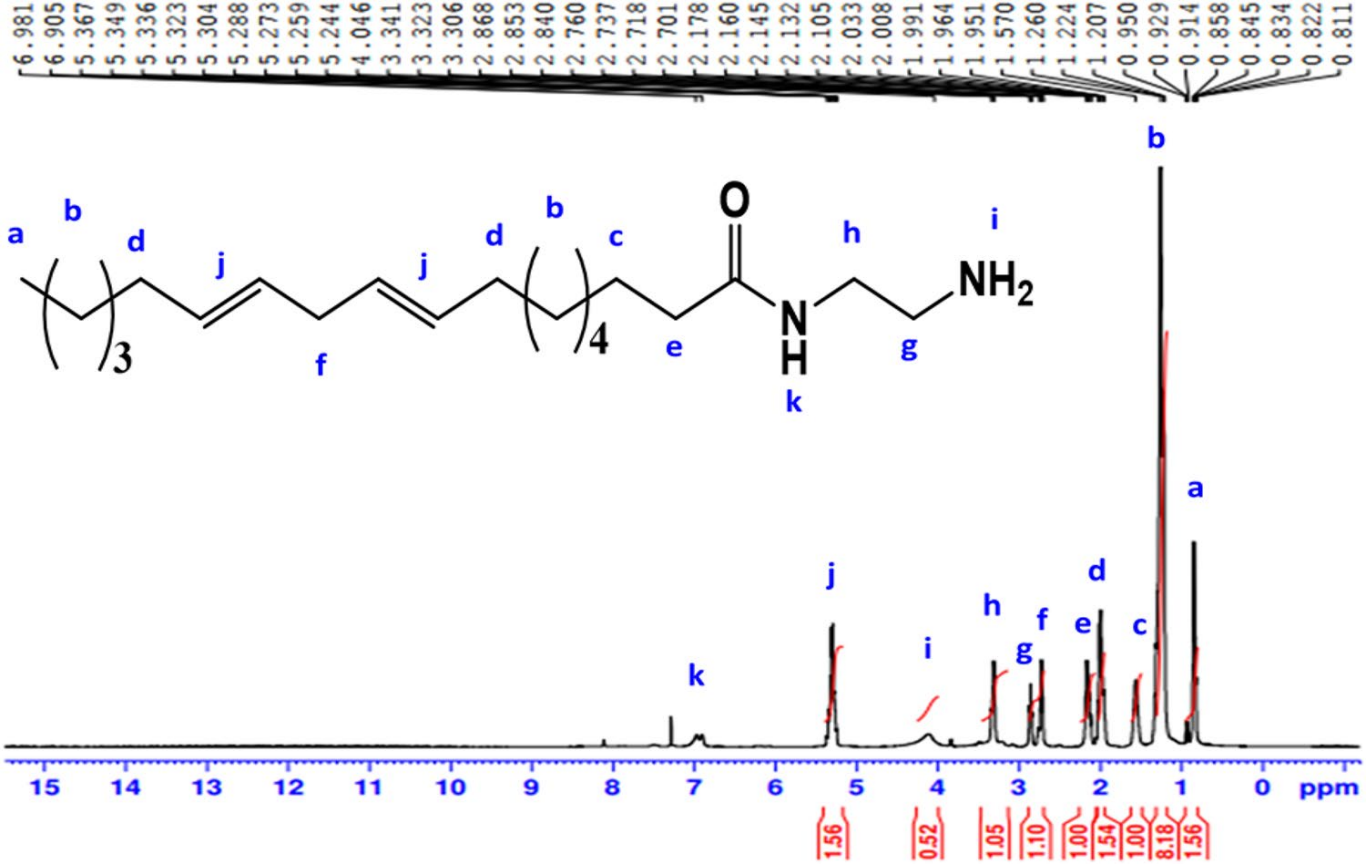
^1^H-NMR spectrum of N-(2-aminoethyl) alkyl amide (AEAA).

^1^H-NMR spectrum (400 MHz, CDCl_3_, δ ppm): of **AEAA** (Fig. 5) demonstrated various peaks at 0.89 (3H, C**H**_**3**_); 1.22 (14H, (C**H**_**2**_)_**7**_); 1.3 (2H, C**H**_**2**_CH_2_CO); 1.57 (4H, C**H**_**2**_CH = CH); 2.03 (2H, C**H**_**2**_C = O); 2.14 (2H, CH = CHC**H**_**2**_CH = CH); 2.84 (2H, NH_2_-C**H**_2_); 3.32 (2H, NHC**H**_**2**_CH_2_); 4.04 (2H, **NH**_**2**_); 5.30 (4H, C**H** = C**H**-CH_2_C**H** = C**H**); 6.98 (1H, N**H**CO).

**Figure 6:**
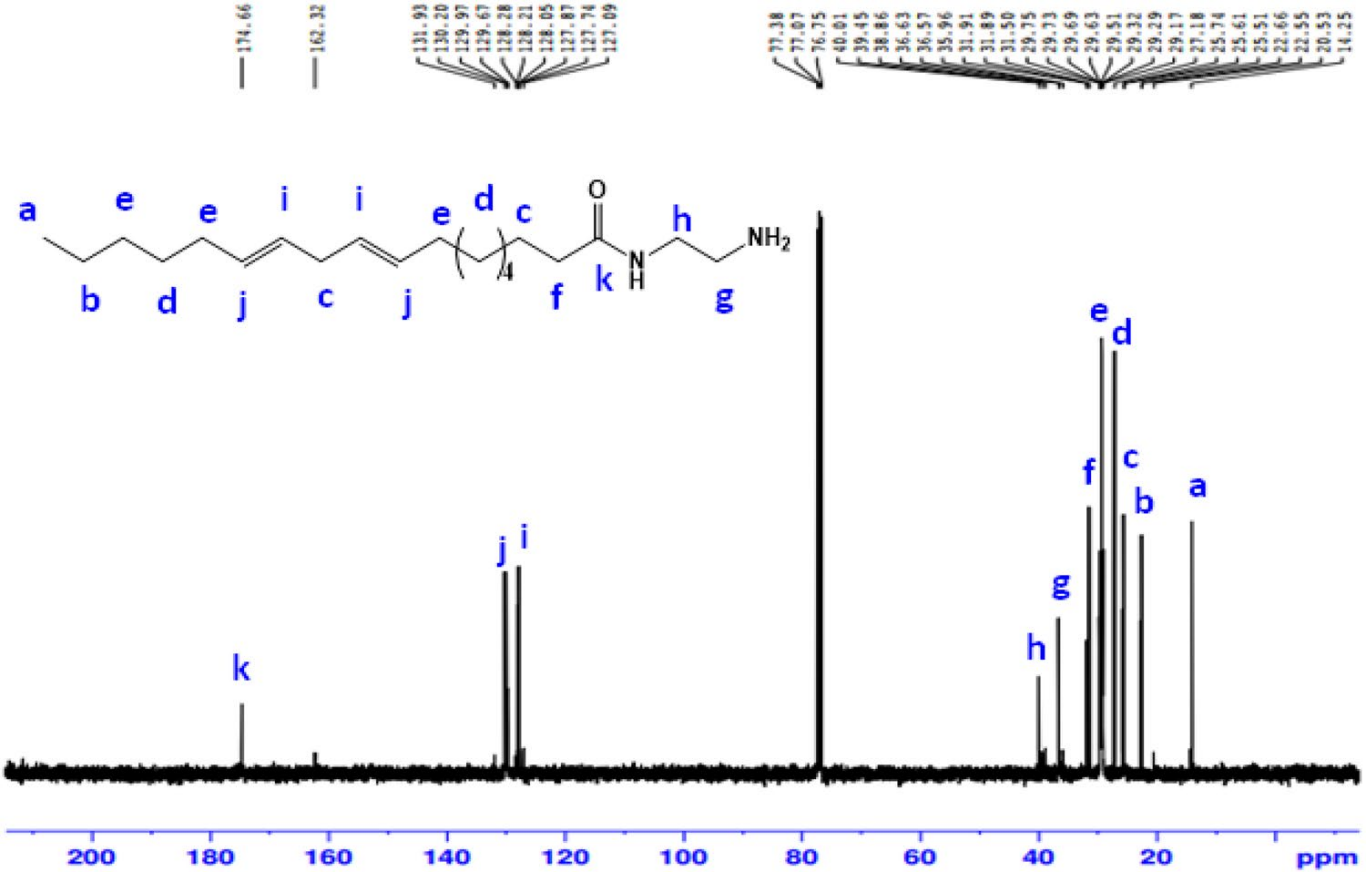
^13^C-NMR spectrum of N-(2-aminoethyl) alkyl amide (AEAA).

The ^13^C-NMR(100 MHz, CDCl_3_, δ ppm ): spectrum of AEAA (Fig. 6) illustrates peaks at 14.25 attributable to carbon of (**C**H_3_); 22.66 (CH_3_**C**H_2_(CH_2_)); 25.61 (**C**H_2_CH_2_CO, (CH = CH**C**H_2_CH = CH); 27.18 ((**C**H_2_)_**4**_CH_2_CH_2_CO, CH = CHCH_2_CH = CHCH_2_**C**H_2_); 29.63 (CH_3_CH_2_**C**H_2_, **C**H_2_CH = CHCH_2_CH = CH**C**H_2_); 31.89 (**C**H_2_C = O); 36.63 (NHCH_2_**C**H_2_NH_2_); 40.01 (NH**C**H_2_CH_2_NH_2_); 128.28 (CH = **C**HCH_2_**C**H = CH); 130.20 (**C**H = CHCH_2_CH = **C**H); 174.66 (**C** = O).

Mass spectrum (Fig. S5) of AEAA exhibited a molecular ion peak [M +] at m/z 322.41 (39.5% C_20_H_38_N_2_O) with a base peak at 55.13 (100%, C_2_H_2_NO), and various fragments of this compound give the peaks with different intensities at different m/z positions as at 321.79 (16%, C_2_HNO), 305.45 (44% C_19_H_35_N_2_O), 71.39 (30% C_5_H_11_), 43.86 (50% C_2_H_6_N), 43.14 (71% CHNO). The mass spectrum analysis affirmed the chemical composition of the synthesized AEAA.

#### Chemical structure of 4-((2-alkylamidoethyl)amino)-4-oxobutanoic acid (AEOB)

FT-IR (KBr, *ν*_max_ cm^−1^) (Fig. S6): 3416.41(ν_O-H_), 3302.75 (*ν*_NH_ stretch), 2922.95, 2851.21 (*ν*_C–H_ asym. and sym. stretch), 1712.89 (*ν*_C=O,_ acid), 1642.23 (*ν*_C=O_, amide), 1555.69 (*ν*_C-N_ stretch).

**Figure 7:**
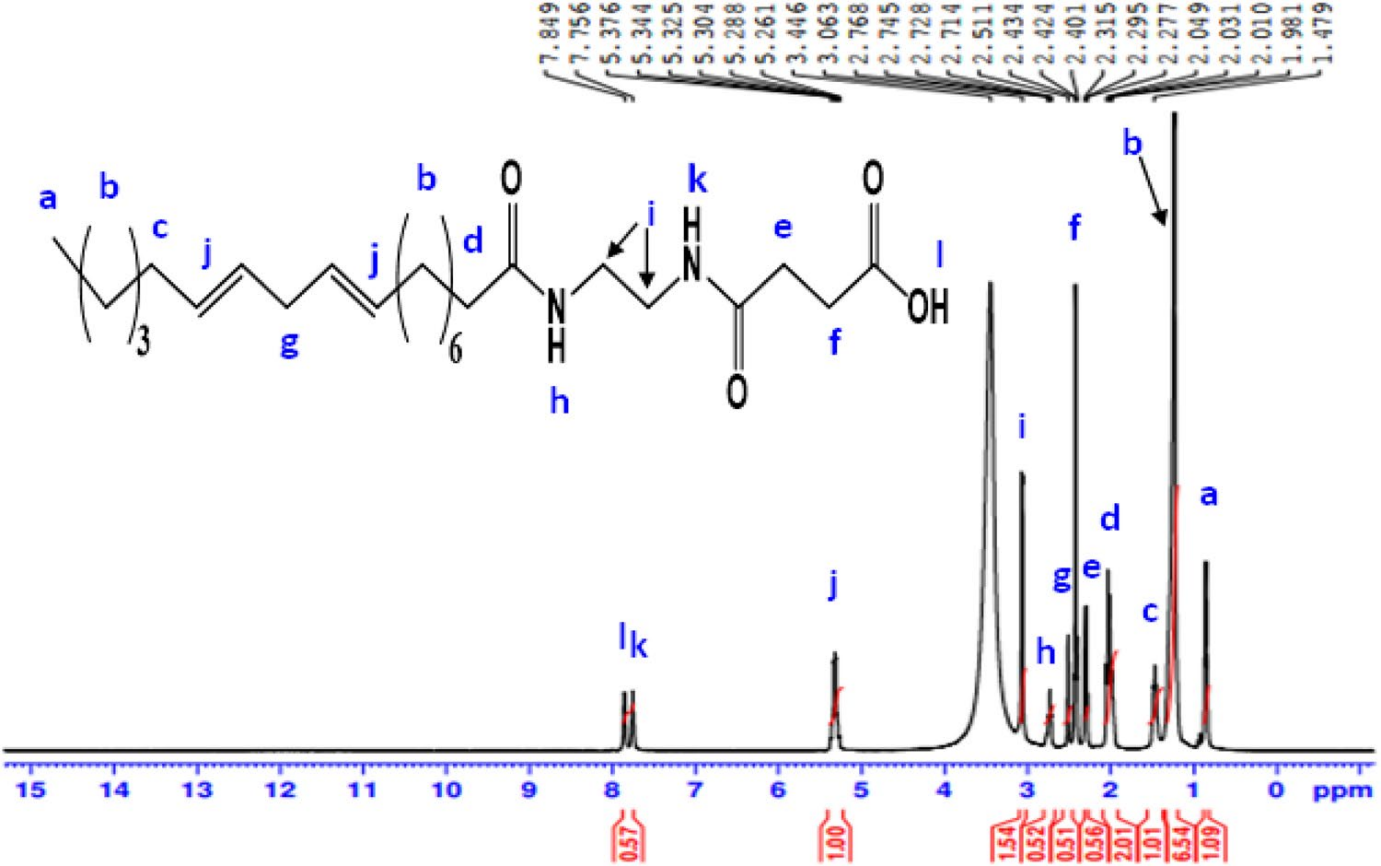
^1^H-NMR spectrum of 4-((2-alkylamidoethyl)amino)-4-oxobutanoic acid (AEOB).

^1^H-NMR spectrum (400 MHz, DMSO, δ ppm): of **AEOB** (Fig. 7) revealed various peaks at 0.89 (3H, C**H**_**3**_); 1.27 (18H, (C**H**_**2**_)_9_); 1.47 (2H, C**H**_**2**_CH = CH); 2.03 (2H, (CH_2_)_6_C**H**_**2**_C = O); 2.29 (2H, C**H**_**2**_CH_2_COOH); 2.43 (2H, CH_2_C**H**_**2**_COOH); 2.51 (2H, CH = CHC**H**_**2**_CH = CH); 2.74 (1H, N**H**CH_2_CH_2_NH); 3.06 (4H, NHC**H**_**2**_C**H**_**2**_NH); 5.32 (4H, C**H** = C**H**CH_2_C**H** = C**H**) ); 7.75 (1H, N**H**COCH_2_CH_2_COOH); 7.84 (1H, COO**H**).

#### Chemical structure of nonionic emulsifiers (NS)

FT-IR (KBr, *ν*_max_ cm^−1^) (Fig. S7): 3423.94(ν_O-H_), 2906.89, 2872.18 (*ν*_C–H_ asym. and sym. stretch), 1249.53, 1731.29 (ν_C−O−C_, *ν*_C=O_, ester), 1649.18 (*ν*_C=O_, amide), 1547.61 (*ν*_C-N_ stretch), 1105.62 (ν_C−O−C_) ethereal band, asym. stretch).

**Figure 8:**
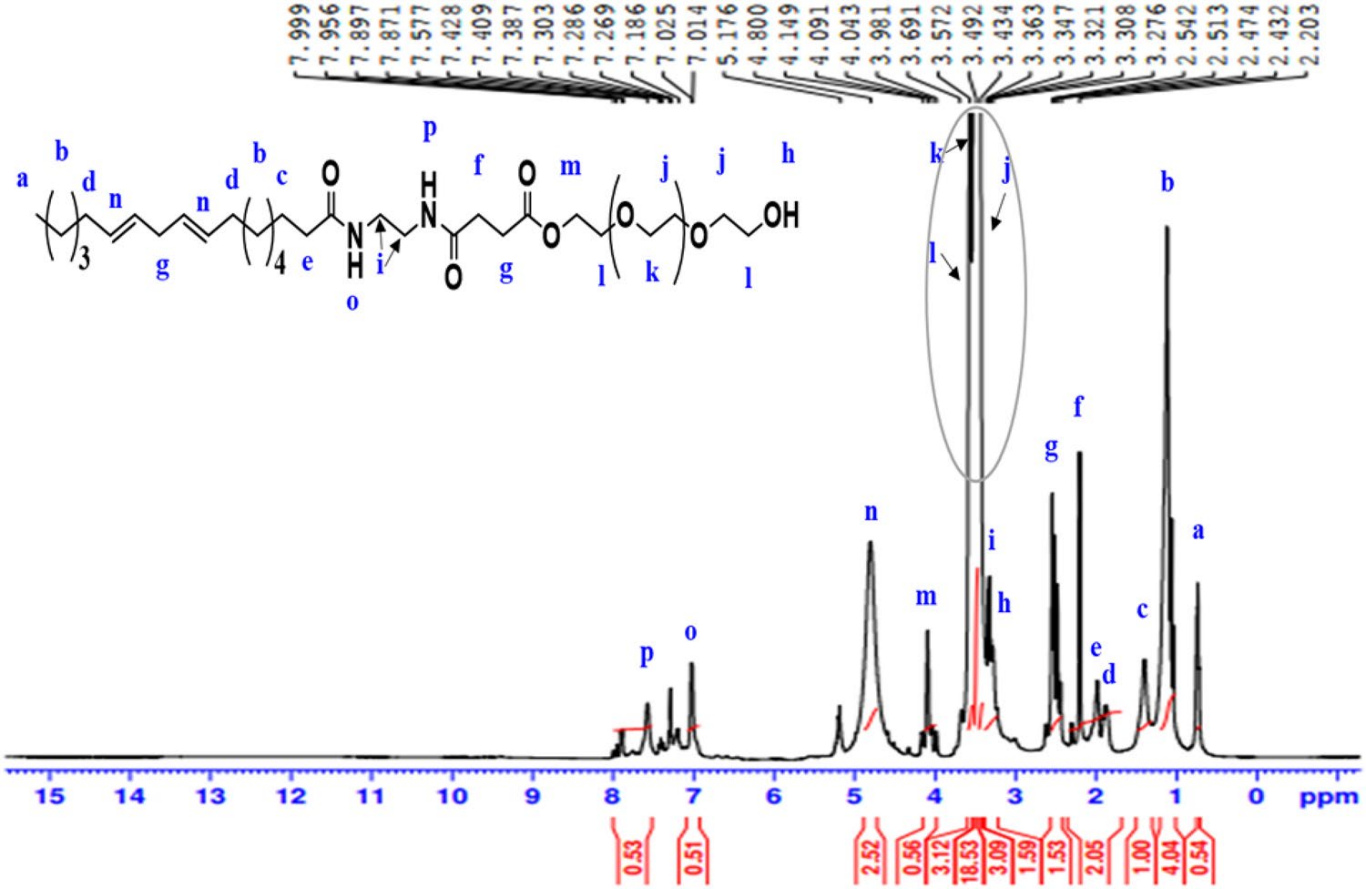
^1^H-NMR spectrum of nonionic emulsifier (NS).

^1^H-NMR spectrum (400 MHz, CDCl_3_, δ ppm ): of **NS** (Fig. 8) revealed various peaks at 0.75 (3H, C**H**_**3**_); 1.12 (14H, (C**H**_**2**_)_**7**_); 1.41 (2H, (CH_2_)_4_C**H**_**2**_CH_2_); 1.88 (4H, (CH_2_)_3_C**H**_**2**_CH = CH, CH = CHC**H**_**2**_(CH_2_)_4_); 1.98 (2H, ((CH_2_)_4_CH_2_C**H**_**2**_); 2.43 (2H, C**H**_**2**_CH_2_COOCH_2_); 2.54 (4H, CH = CHC**H**_**2**_CH = CH, CH_2_C**H**_**2**_COOCH_2_); 3.28 (1H, OH); 3.34 (4H, NHC**H**_**2**_C**H**_**2**_NH); 3.45 (4H, C**H**_**2**_CH_2_OH, (OCH_2_C**H**_**2**_)); 3.56 (2H, (OC**H**_**2**_CH_2_)); 3.67 (2H, (CH_2_C**H**_**2**_OH), 3.68 (2H, COOCH_2_C**H**_**2**_); 4.10 (2H, COOC**H**_**2**_CH_2_); 4.80 (4H, C**H** = C**H**CH_2_C**H** = C**H**); 7.03 (1H, (CH_2_)_6_CON**H**); 7.57 (1H, N**H**COCH_2_CH_2_).

### Chemical structure confirmation of synthesized mono and gemini cationic surfactants based on recycled vegetable oil

The synthesis of mono and gemini cationic surfactants as follows. N-(2-aminoethyl)alkyl amide (AEAA) is dissolved in dichloromethane and mixed with aqueous solution of Na_2_CO_3_ at 5℃. Then, chloroacetyl chloride is added to the cooled mixture and stirred at room temperature for 2 h, resulting in the formation of N-(2-alkylamidoethyl)-2-chloroacetamide (AECA)^43^. Subsequently, AECA reacts with a solution of triethylamine and N,N,N′,N′-Tetramethylethane-1,2-diamine in ethyl acetate, leading to the formation of mono and gemini cationic surfactants, respectively^44,45^. The chemical reaction was illustrated in Fig. (S1).

#### Chemical structure of N-(2-alkylamidoethyl)-2-chloroacetamide (AECA)

FT-IR (KBr, *ν*_max_ cm^−1^) (Fig. S8): 3284.76 (*ν*_NH_ stretch), 2922.51, 2852.38 (*ν*_C–H_ asym. and sym. stretch), 1638.02 (*ν*_C=O_, amide), 1555.87 (*ν*_C-N_ stretch).

^1^H-NMR spectrum (400 MHz, CDCl_3_, δ ppm ): of **AECA** (Fig. S9) demonstrated various peak at 0.90 (3H, C**H**_**3**_); 1.30 (14H, (C**H**_**2**_)_**7**_); 1.58 (2H, C**H**_2_CH_2_CO); 2.01 (4H, C**H**_**2**_CH = CH); 2.18 (2H, C**H**_**2**_C = O); 2.76 (2H, CH = CHC**H**_**2**_CH = CH); 3.40 (2H, NHC**H**_**2**_CH_2_NHCO); 4.00 (2H, C**H**_**2**_NHCOCH_2_Cl); 5.36 (4H, C**H** = C**H**-CH_2_C**H** = C**H** ); 6.59 (2H, C**H**_**2**_Cl); 7.53 (1H, CH_2_CH_2_CON**H**); 8.14 (1H, N**H**COCH_2_Cl).

#### Chemical structure of 2-((2-alkylamidoethyl)amino)-N,N,N-triethyl-2- oxoethan-1-aminium chloride (MCS)

FT-IR (KBr, *ν*_max_ cm^−1^) (Fig. S10): 3305.73 (*ν*_NH_ stretch), 2921.04, 2851.38(*ν*_C–H_ asym. and sym. stretch), 1640.69 (*ν*_C=O_, amide), 1555.56 (*ν*_C-N_ stretch).

**Figure 9:**
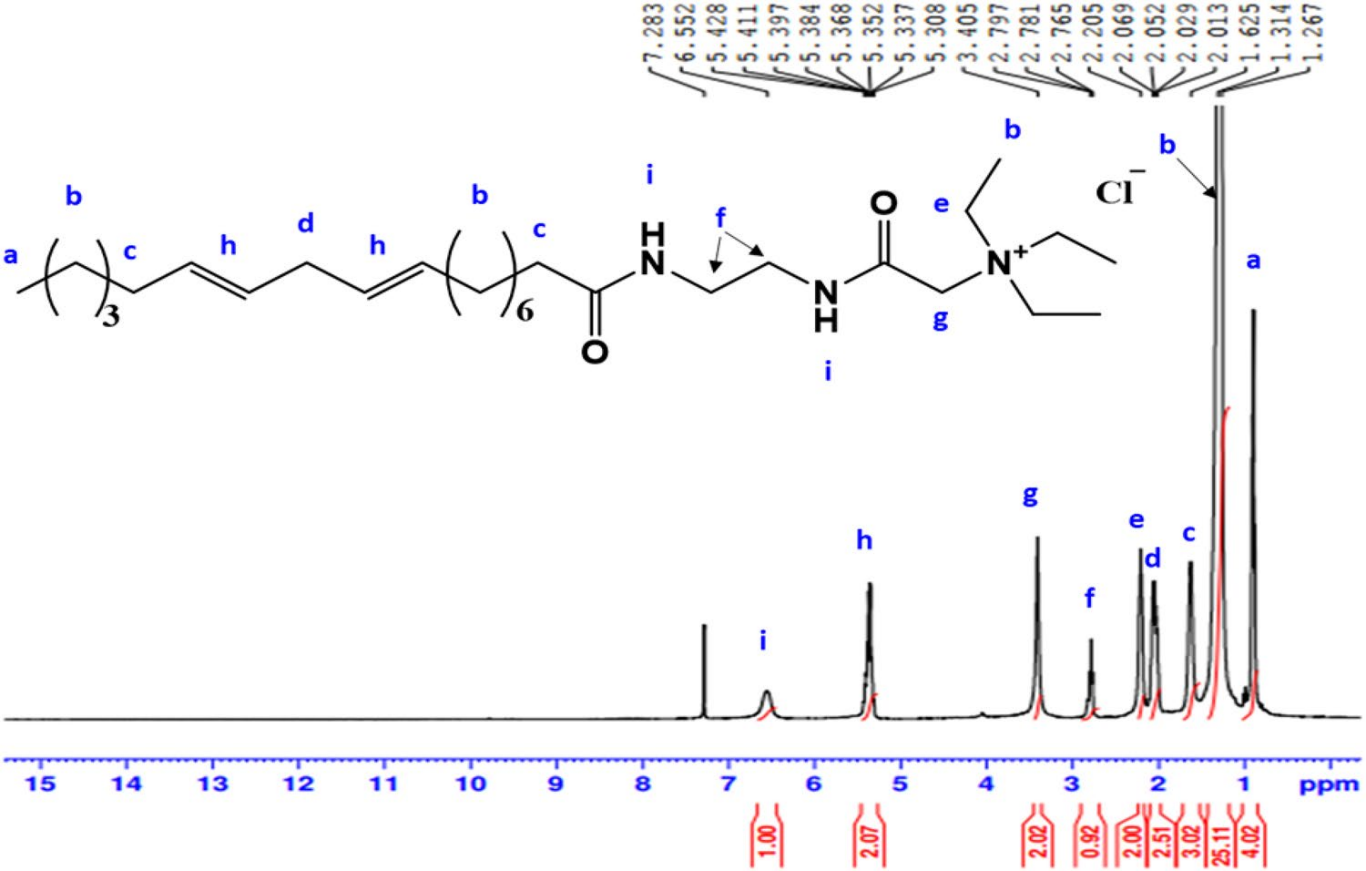
^1^H-NMR spectrum of 2-((2-alkylamidoethyl)amino)-N,N,N-triethyl-2-oxoethan-1-aminium chloride (MCS).

^1^H-NMR spectrum (400 MHz, CDCl_3_, δ ppm ): of **MCS** (Fig. 9) revealed various peaks at 0.98 (3H, C**H**_**3**_); 1.31 (27H, (C**H**_**2**_)_9_), ((C**H**_**3**_CH_2_)_3_N^**+**^); 1.62 (4H, C**H**_**2**_CH = CH, ((CH_2_)_6_C**H**_**2**_C = O); 2.06 (2H, CH = CHC**H**_**2**_CH = CH); 2.20 (6H, N^**+**^(C**H**_**2**_CH_3_)_3_); 2.78 (4H, NHC**H**_**2**_C**H**_**2**_); 3.40 (2H, COC**H**_**2**_N^**+**^); 5.39 (4H, C**H** = C**H**CH_2_C**H** = C**H**); 6.52 (2H, N**H**CO).

#### Chemical structure of N,N′-bis(2-((2-alkylamidoethyl)amino)-2-oxoethyl)- N,N,N′,N tetramethylethane-1,2-diaminium chloride (GCS)

FT-IR (KBr, *ν*_max_ cm^−1^) (Fig. S11): 3276.88 (*ν*_NH_ stretch), 2924.37, 2852.84 (*ν*_C–H_ asym. and sym. stretch), 1641.48 (*ν*_C=O_, amide), 1548.91 (*ν*_C-N_ stretch).

**Figure 10:**
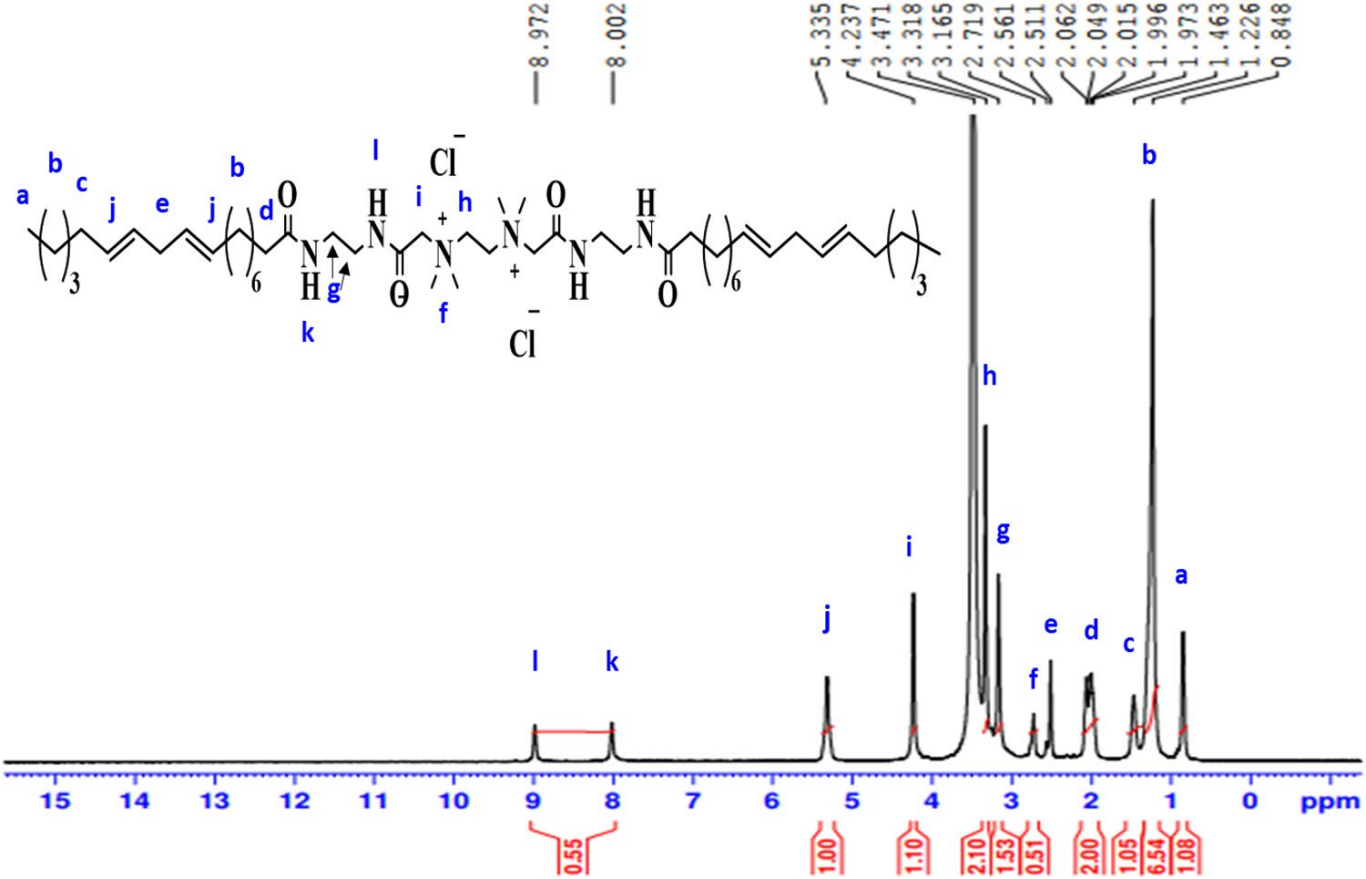
^1^H-NMR spectrum of N,N′-bis(2-((2-alkylamidoethyl)amino)-2-oxoethyl)-N,N,N′,N tetramethylethane-1,2-diaminium chloride (GCS).

^1^H-NMR spectrum (400 MHz, DMSO, δ ppm ): of **GCS** (Fig. 10) revealed various peaks at 0.84 (3H, C**H**_**3**_); 1.22 (18H, (C**H**_**2**_)_9_); 1.46 (2H, C**H**_**2**_CH = CH); 2.04 (2H, (CH_2_)_6_C**H**_**2**_C = O); 2.56 (2H, CH = CHC**H**_**2**_CH = CH); 2.71 (6H, (C**H**_**3**_)_**2**_NCH_2_); 3.16 (4H, NHC**H**_**2**_C**H**_**2**_NH); 3.31 (2H, (CH_3_)_2_N^**+**^C**H**_**2**_); 4.23 (2H, COC**H**_**2**_ N^**+**^(CH_3_)_2_); 5.33 (4H, C**H** = C**H**-CH_2_C**H** = C**H**): 8.00 (1H, N**H**CH_2_CH_2_NH); 8.97 (1H, N**H**COCH_2_N(CH_3_)_2_).

### Antimicrobial activity of mono and gemini cationic surfactants

Metal cutting fluids may become contaminated by microorganisms from a variety of sources, including contaminated raw materials, improper cleaning techniques, airborne contamination, human contact, mixing with water, and system leaks. To limit microbial growth, biocides must be added to metal cutting fluid^56,57^.

Cationic surfactants have good resistance towards microbial activity due to their positive charge which has a strong affinity for negatively charged the microbial cell membrane. Furthermore, the presence of these surfactants' long hydrophobic alkyl chains can interact with the hydrophobic lipid membrane. In addition, the counter anion may penetrate the cell's cytoplasm. These effects cause the cell's selective permeability to be disrupted, resulting in cell death^58–60^.

In vitro results of antibacterial and antifungal activity of synthesized mono and gemini cationic surfactants are reported in (Table 2) for various microbes called *Bacillus pumilis, Streptococcus faecalis, Escherichia coli, Enterobacter cloacae, Saccharomyces cerevisiae,* and *Candida albicans* compared to Penicillin G, Ciprofloxacin, and Ketoconazole-respectively. The antimicrobial activity of the surfactants studied was first evaluated using the disc diffusion method. All of the microorganisms studied were inhibited by the solutions of each surfactant applied to the plates. All of the surfactants tested had inhibition zones ranging from 10 to 20 mm for all types of bacteria and fungi species, which were termed active^61^. The GCS and MCS displayed the highest anti-bacterial behavior (20, 19, and 18,15 mm) to *E.coli* and *B. pumilis* relative to the control (28, 26 mm) respectively. Whereas, the GCS and MCS displayed the highest anti-fungal behavior (15, 16 mm) to *C. albicans* compared to control (24 mm) respectively. According to the results, the tested compounds have significant anti-bacterial and anti-fungal activities towards all tested microorganisms, with the GCS having better anti-bacterial activity. Gemini cationic surfactants differ from mono-cationic surfactants in their molecular structure, where they have two positively charged groups instead of one. Because of this dual charge, gemini surfactants adsorb more strongly to negatively charged bacterial cell walls, disrupting the cell membrane and causing cell death^58–60^.

**Table 2:** Antimicrobial activity of synthesized MCS & GCS against Gram-positive, Gram-negative bacteria and Fungi.

Sample code	Gram ( +) Bacteria		Gram (-) Bacteria		Fungi	
	*B. pumilis*	*S. faecalis*	*E.Coli*	*E. cloacae*	*S. cerevisiae*	*C. albicans*
Inhibition zone(mm)						
GCS	18	17	20	17	12	15
MCS	15	15	19	18	10	16
Penicillin G	26	23	–	–	–	–
Ciprofloxacin	–	–	28	26	–	–
Ketoconazole	–	–	–	–	20	24

Gemini outperformed mono-cationic surfactants in terms of broad antibacterial activity against both Gram-positive and Gram-negative organisms. Therefore, the antimicrobial activity of the gemini cationic compound was quantitatively evaluated by measuring the minimum inhibitory concentrations (MICs), and the minimum bacterial concentration (MBC), for *B. pumilis*, *S. faecalis, E. coli,* and *E. Cloacae* using the micro broth dilution method. Remarkably, GCS had the lowest MIC and MBC values against *E.coli* (3.91, 15.6 g/ml, respectively) as shown in Table 3. It implies that the GCS has potent antibacterial activity and is used in MCF formulations.

**Table 3:** The minimum inhibitory concentrations (MIC), and the minimum bacterial concentration (MBC) of GCS.

Sample code	MIC (µg/ml)				MBC (µg/ml)			
	*B. pumilis*	*S. faecalis*	*E. coli*	*E. cloacae*	*B. pumilis*	*S. faecalis*	*E. coli*	*E. cloacae*
GCS	15.6	31.3	3.91	7.81	31.3	62.5	15.6	62.5

### Formulation of metal cutting fluids (cutting fluid emulsions or emulsifiable soluble oils)

Emulsifiable soluble oils are formulated by blending vegetable base oil with emulsifying agents (individual or mixed emulsifiers), lubricant oil (oiliness), biocide, corrosion inhibitor, and coupling agent^35^. This cutting oil package has been diluted with water to create an oil-in-water emulsion. The resulting fluid is milky white in color. The distinctive advantage of this kind of fluid is that it combines the cooling properties of water and the lubricating properties of oil^62,63^. Vegetable oil's highly lubricating properties can be attributed to the fundamental chemical composition of vegetable oil molecules (it contains a polar group). Where the polar group draws the vegetable oil molecule to a metallic surface creating a thick, strong, and durable lubricant film that reduces friction between the cutting tool and the workpiece during machining operations^64^.

The emulsifying agent plays a crucial role in stabilizing the cutting fluid emulsion by reducing the interfacial tension between the oil and water, forming a protective layer around each oil droplet, and reducing the droplet size, which prevents coalescence and separation of the oil from the water^65^. All cutting fluid emulsion formulations include a corrosion inhibitor to prevent rusting and corrosion on metal surfaces. They act by creating a layer of protection on the metal's surface. The corrosion inhibitor can help to increase cutting efficiency, decrease maintenance costs, and lengthen the life of cutting tools^21^. Biocide is applied in cutting fluid emulsion formulations to inhibit the growth of microorganisms, which can lead to a variety of issues, including corrosion, odor, health risks, and decreased performance. Biocides are added in low concentrations to ensure their effectiveness and to minimize any potential negative effects on the cutting fluid itself^63,66^. Additionally, oiliness plays a role in providing better lubrication and improving the workpiece surface finish^21,67^. Finally, coupling agents are incorporated into every formulation to improve the compatibility and solubility of additives in the oil, resulting in enhanced performance and stability of the fluid^63^.

### Evaluation methods of different cutting fluid emulsions or emulsifiable soluble oils formulas using individual emulsifiers

#### Cutting oil package stability

**Figure 11:**
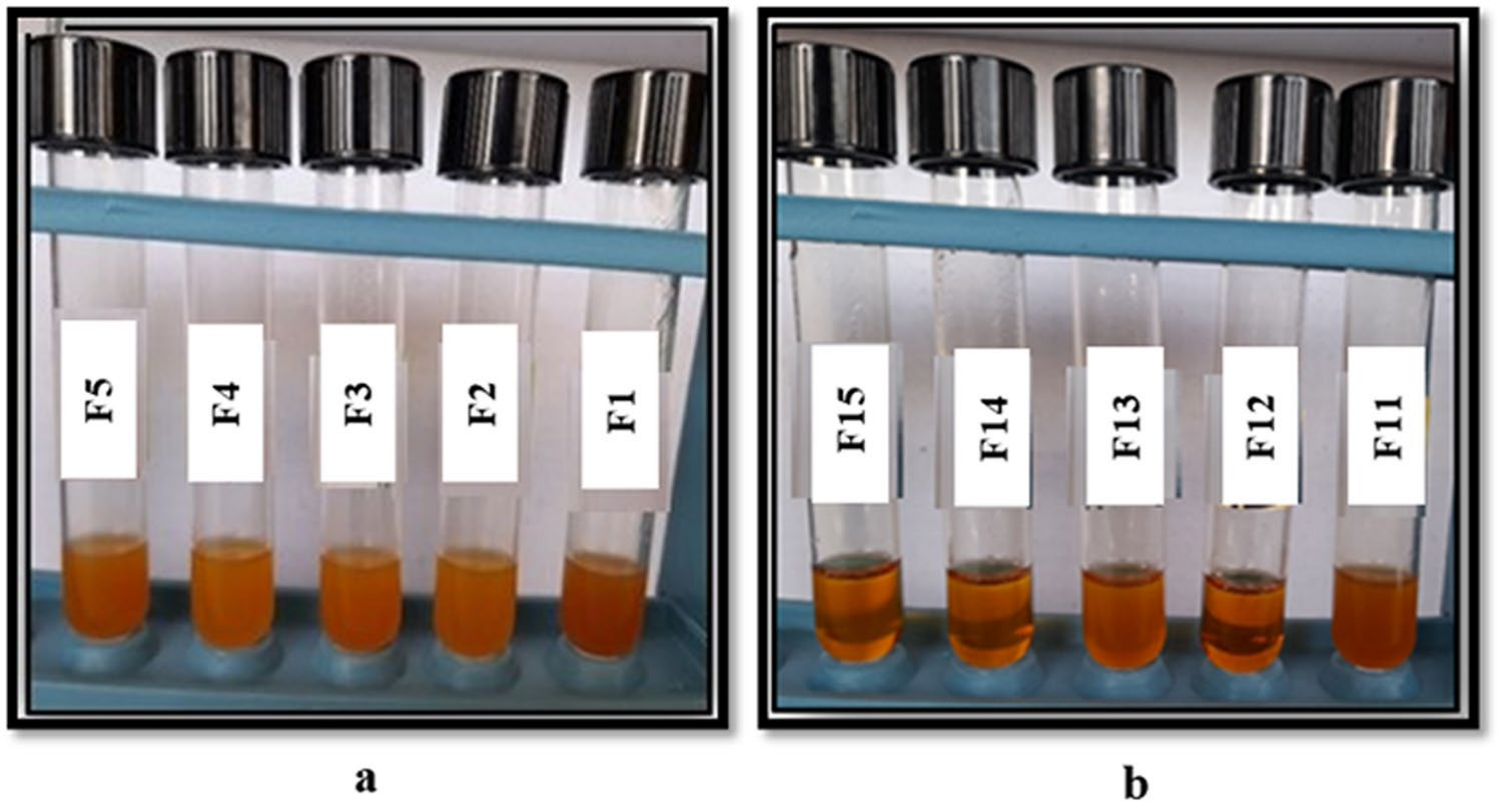
(**a**,**b**): Some representative photos of cutting oil packages using individual emulsifiers at different concentrations; (**a**) anionic and (**b**) nonionic emulsifiers.

To achieve optimum cutting oil package stability, a series of cutting oil formulas with different ingredient ratios were created. A formula that gives a stable cutting oil package of homogeneous clear oil (without separation or indications of turbidity), as shown in Fig. 11 (a, b) was chosen for additional studies.

#### Emulsion stability of emulsifiable soluble oils

**Figure 12:**
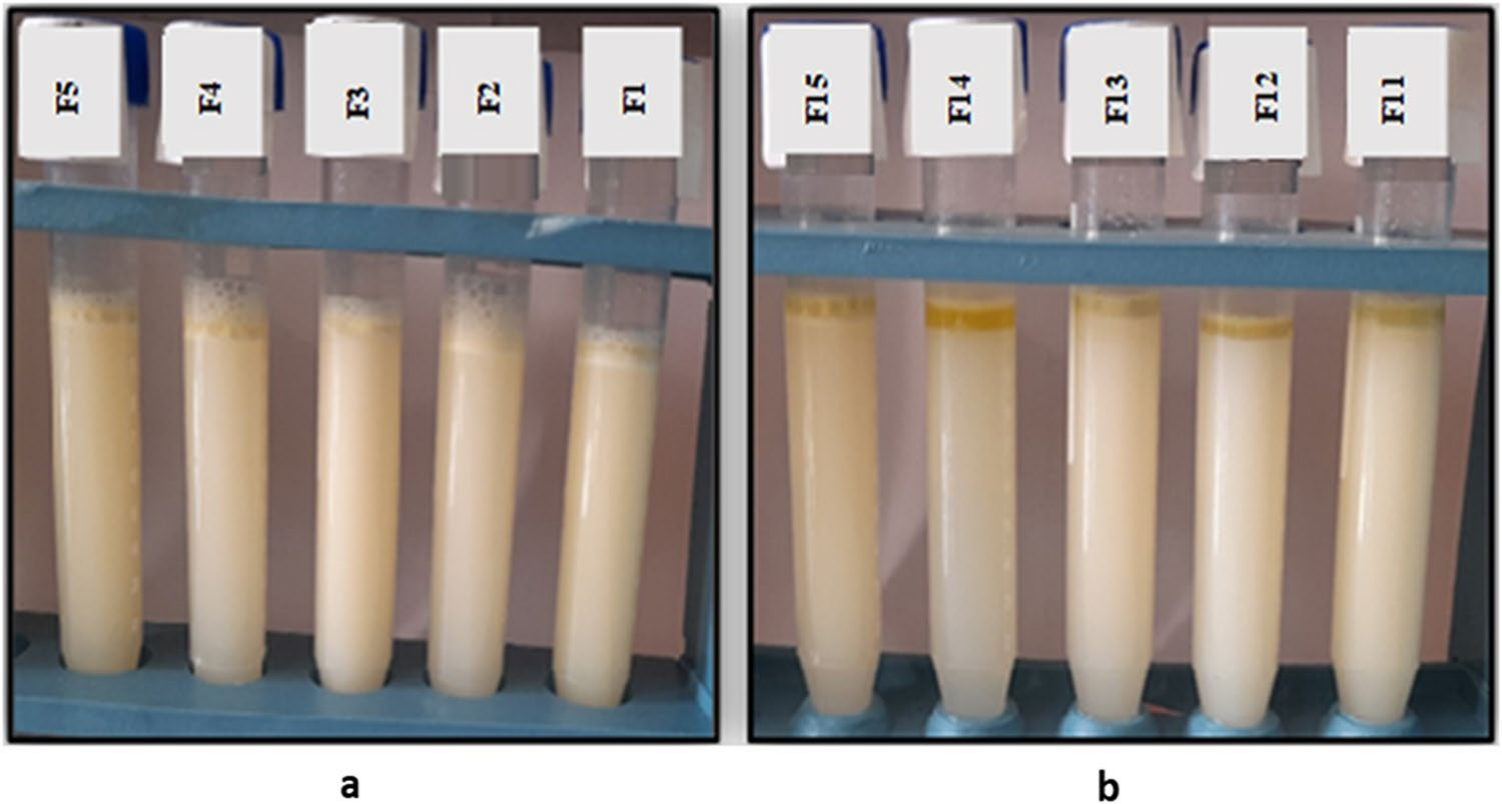
(**a**,**b**): Some representative photos of emulsion stability using individual emulsifiers at different concentrations; (**a**) anionic and (**b**) nonionic emulsifiers.

Emulsifiable soluble oils are typically made by dispersing a cutting oil package in tap water in a volume ratio of 5/95. After 24 h of preparation, the stability of these emulsions was observed. The concentration of the incorporated components in all formulations has an impact on the metal cutting emulsion's stability and performance. It is clear from Fig. 11 (a, b) that the cutting oil formulas provide oil stability, but they are unstable and result in oil separation or gel formation in the emulsion stability test, as shown in Fig. 12 (a, b).

### Formulation of different cutting fluid emulsions or emulsifiable soluble oils using mixture emulsifiers

By utilizing a blend of two or more diverse emulsifiers, rather than individual emulsifiers, the formation, stability, and performance of oil-in-water emulsions can be significantly improved^63,68^. Mixed emulsifiers exhibit diverse structures and properties (Mixed emulsifiers can have a variety of different structures and properties), influencing their capacity to form stable emulsions. For instance, one type of emulsifier may exhibit rapid adsorption and effective reduction of interfacial tension, but may not be as proficient at preventing droplet coalescence. Conversely, another emulsifier may not be as efficient in reducing interfacial tension but is highly efficient in inhibiting droplet coalescence. By combining emulsifiers with varying properties, it becomes possible to optimize the balance of interfacial forces and create a more stable emulsion. Consequently, mixed emulsifiers may have synergistic effects on emulsion stability^68^. Furthermore, the combination of a water-soluble emulsifier and an oil-soluble emulsifier in all cutting fluid formulations can have a synergistic effect on emulsion stability in terms of coalescence rates^69^. Therefore, the HLB of the mixed emulsifiers was calculated. The hydrophilic-lipophilic balance (HLB) value of a surfactant is typically used to express the surfactant's capacity to create an emulsion. Also, it plays a crucial role in emulsion stability^70,71^.

For mixed surfactants, an effective HLB can be calculated using mass percentages and HLB numbers of the individual emulsifiers. The mixed HLB values were calculated by the following equation^21,72^:

1 $${\mathbf{HLB}}_{{{\mathbf{AB}}}} = \, \left( {{\mathbf{HLB}}_{{\mathbf{A}}} * \, \left( {\mathbf{A}} \right) \, \% } \right) \, + \, \left( {{\mathbf{HLB}}_{{\mathbf{B}}} * \, \left( {\mathbf{B}} \right) \, \% } \right)$$

where HLB_AB_, HLB_A,_ and HLB_B_ are HLB of mixed emulsifiers and individual emulsifiers respectively, A% and B% are the mass percentages of A and B in the mixed emulsifiers, respectively.

If the hydrophilic part of the emulsifier has only ethylene oxide, the HLB can be calculated for individual emulsifiers using the following formula^73^.

2 $${\mathbf{HLB}} \, = \, {\mathbf{E}}/{\mathbf{5}}$$

where E denotes the percentage of ethylene oxide molecules in the surfactant molecules.

To determine the optimum HLB value that gives a stable oil-in-water emulsion, a mixture of oil-soluble nonionic and water-soluble nonionic with different hydrophilic-lipophilic balance (HLB) values of 12, 11, 10, 9, and 8 was prepared. It is worth noting that the HLB value of 10 provided the best stability for O/W emulsions for nonionic/nonionic.

### Evaluation methods of different cutting fluid emulsions or emulsifiable soluble oils formulas using mixture emulsifiers

#### Cutting oil package stability

**Figure 13:**
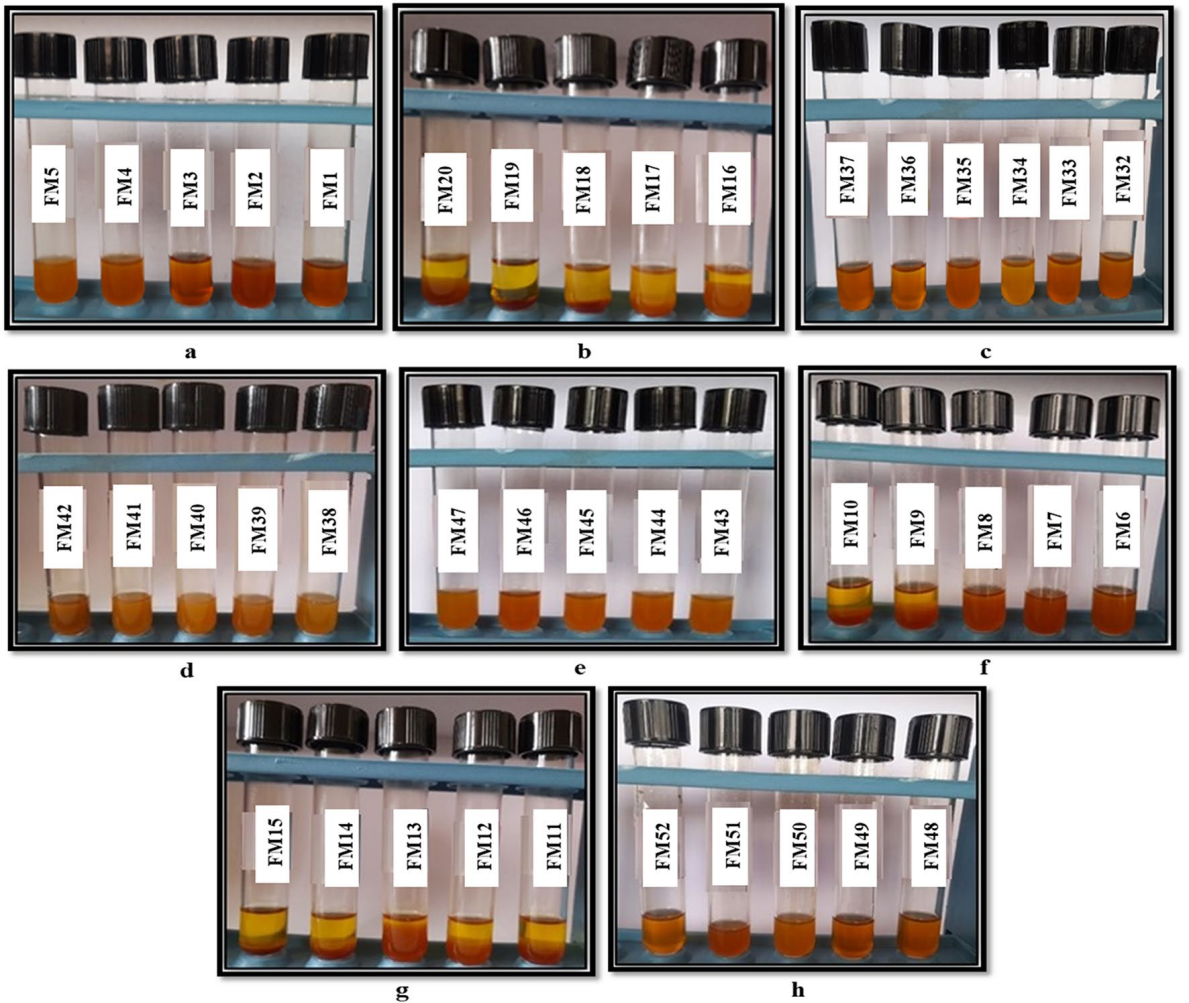
(**a**–**h**): Some representative photos of cutting oil packages using mixed emulsifiers at different concentrations (**a**–**e**) nonionic/nonionic, (**f**–**h**) anionic/nonionic.

A number of cutting oil package formulations with different constituent ratios have been prepared. Figure 13 (a–h), shows the cutting oil package stability of mixed emulsifiers at different ratios of nonionic/nonionic and anionic/nonionic mixture. Some formulas that produced separation in cutting oil package stability will be excluded from the study. While, the formulas that produce stable cutting oil packages with clear, homogeneous oil exhibiting no signs of separation or turbidity were chosen for further investigation.

#### Emulsion stability of cutting oil packages

**Figure 14:**
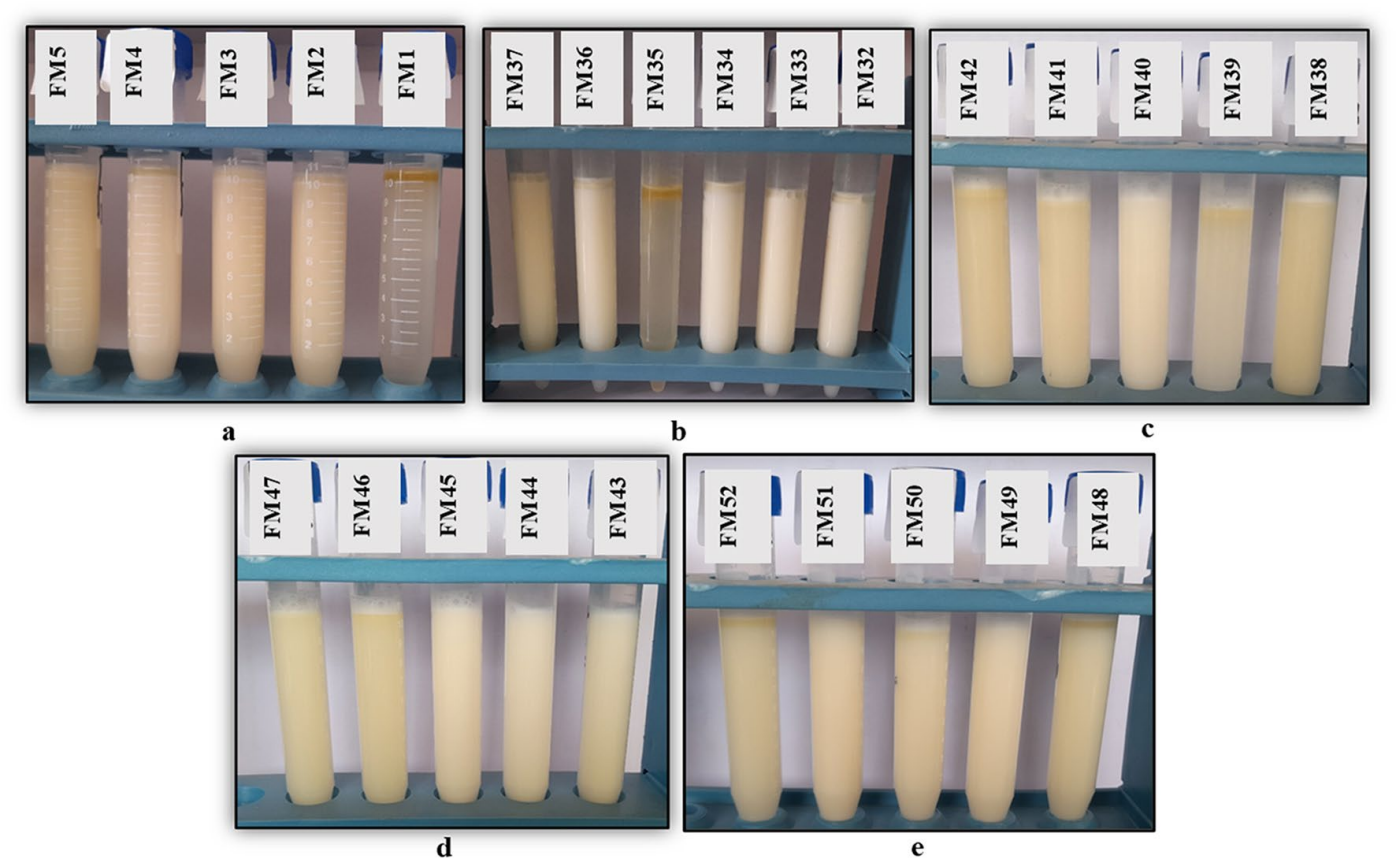
(**a**–**e**): Some representative photos of emulsion stability using mixed emulsifiers at different concentrations (**a**–**d**) nonionic/nonionic, (**e**) anionic/nonionic.

The cutting fluid emulsion was tested for stability over a period of 24 h. Typically, cutting oil packages are dissolved in tap water at a volume ratio of 5/95 to produce emulsifiable soluble oils. The stability and effectiveness of the metal-cutting emulsion are influenced by the concentration of the incorporated ingredients in all formulations. From the results illustrated in Table 4, for emulsifier blends (nonionic/nonionic), blend numbers 32 (Formula I), blend number 43 (Formula II) ), blend number 44 (Formula III) ), blend number 45 (Formula IV) ), for emulsifier blends (anionic/nonionic), blend number 49 (Formula V) and blend number 51 (Formula VI) display the highest emulsion stability as shown in Fig. 14 (a–e). The results of the test revealed that the mixed emulsifiers at different nonionic/nonionic ratios with HLB value 10 (Formulas I, II, III, and IV) and anionic/nonionic ratios (Formulas V and VI) produced stable emulsions. The combination of emulsifier blends can lead to improved emulsion stability through synergistic effects compared to using a single emulsifier alone. Other blends show oil separation or gel formation at the top and also water phase separation is observed at the bottom of test tubes as shown in Fig. 14 (a–e). Formulas I and VI were chosen for additional testing.

**Table 4:** Formulation of different cutting fluid emulsions using mixture emulsifiers.

Ingredients	Formula I	Formula II	Formula III	Formula IV	Formula V	Formula VI
Recycled vegetable oil	80	82	82	82	80	80
Emulsifier mixture						
Sodium 2-alkylamidoethyl sulfate (AS)					6	10
2-(2-hydroxyethoxy)ethyl 4-((2-((9E,12E)-octadeca-9,12-dienamido)ethyl)amino)-4-oxobutanoate (NS1)	3.927	3.93	2.29	2.29		2
38-hydroxy-3,6,9,12,15,18,21,24,27,30,33,36-dodecaoxaoctatriacontyl 4-((2-((9E,12E)-octadeca-9,12-dienamido)ethyl)amino)-4-oxobutanoate (NS3)			7.71	7.71	6	
65-hydroxy-3,6,9,12,15,18,21,24,27,30,33,36,39,42,45,48,51,54,57,60,63-henicosaoxapentahexacontyl 4-((2-((9E,12E)-octadeca-9,12-dienamido)ethyl)amino)-4-oxobutanoate (NS4)	6.073	6.07				
N,N′-bis(2-((2-alkylamidoethyl)amino)-2-oxoethyl)-N,N,N′,N tetramethylethane-1,2-diaminium chloride (GCS as biocide and corrosion)	3	2	2	1	1	1
Oleic acid (oiliness)	4	3	3	4	4	4
Diethyleneglycol (coupling agent)	3	3	3	3	3	3

#### Corrosion inhibition test

**Figure 15:**
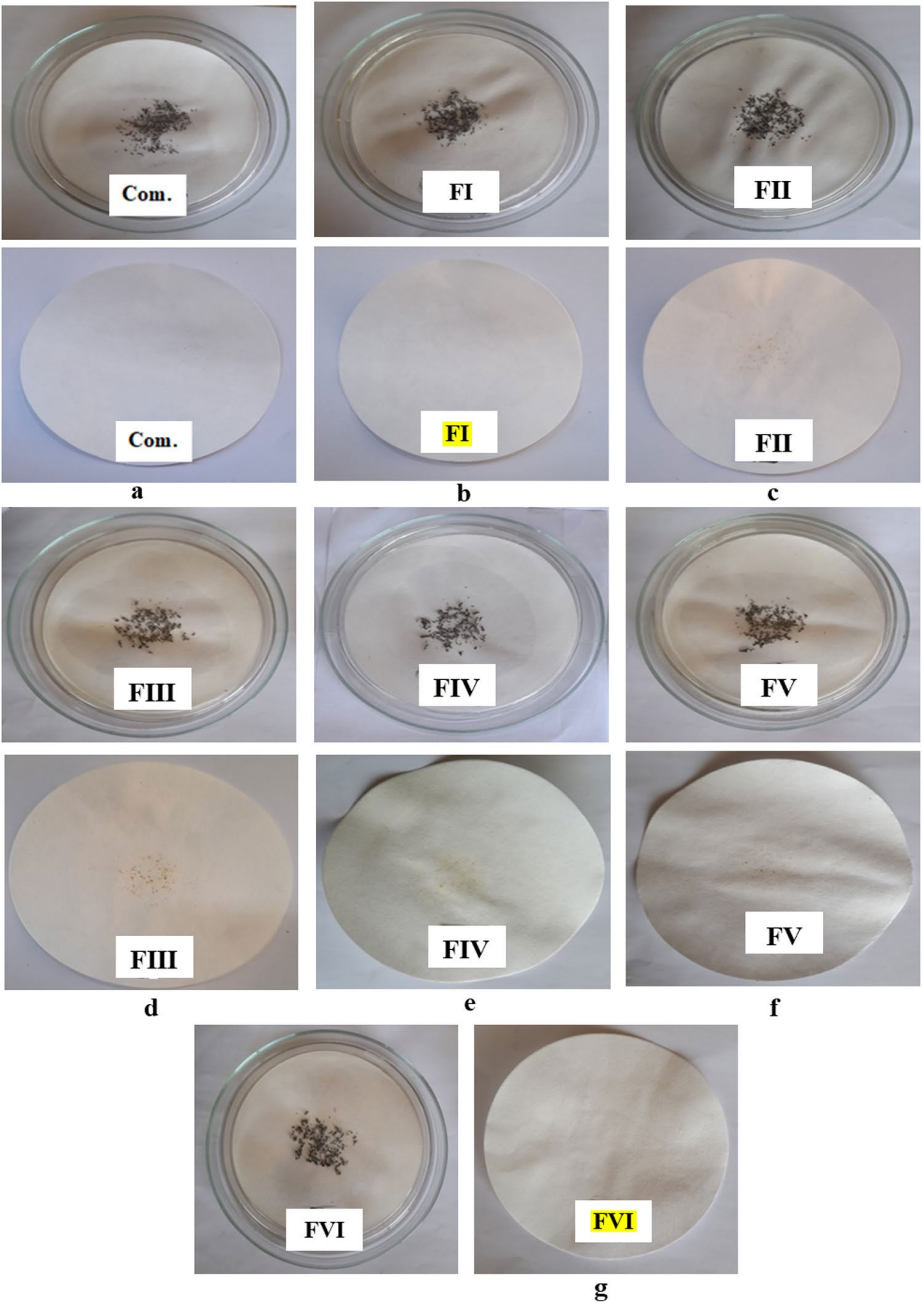
(**a**–**g**): Anti-corrosion test for (**a**) commercial sample, (**b**–**e**) nonionic/nonionic(FI-FIV), (**f**,**g**) anionic/nonionic(FV, FVI) N.B.  best formula in corrosion inhibtion test.

A corrosion test was performed for all stable soluble oil emulsions (FI-FVI and commercial samples). Figure 15 (a–g) depicts the corrosion test results for Formulas I, VI, and the commercial sample, which displayed good protection effectiveness. Many rust spots and stains on the filter papers appeared for formulas (FII-FV) (see Fig. 15 (c–f)). Formulas I and VI were chosen for further investigation.

#### Wettability test (contact angle)

Wettability plays a crucial role in metal-cutting fluids and the behavior of liquids on solid substrates. Wettability refers to the ability of a liquid to spread or adhere to a solid surface^74^. The contact angle is a key parameter used to quantify wettability. The contact angle of a cutting fluid on a metal surface can significantly impact its effectiveness during metal cutting. A low contact angle indicates good wetting and spreading of the cutting fluid on the metal surface. This allows the fluid to form a continuous and uniform film, providing efficient cooling and lubrication^75^. The Young equation can be stated as follows:

3 $${{\varvec{\upgamma}}}\__{{{\mathbf{solid}}\_{\mathbf{liquid}}}} \; = \;{{\varvec{\upgamma}}}\__{{{\mathbf{solid}}\_{\mathbf{gas}}}} \; + \;{{\varvec{\upgamma}}}\__{{{\mathbf{liquid}}\_{\mathbf{gas}}*}} \;{\mathbf{cos}}\left( {{\varvec{\uptheta}}} \right)$$

where: γ_solid_liquid is the interfacial tension between the solid and liquid phases, γ_solid_gas is the interfacial tension between the solid and gas phases, γ_liquid_gas is the interfacial tension between the liquid and gas phases, and *θ* is the contact angle.

**Figure 16:**
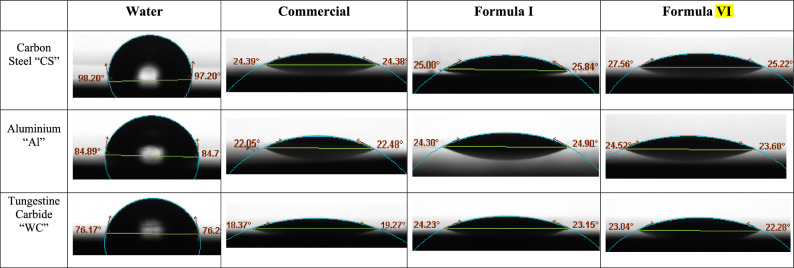
Contact angle of water, commercial sample, FI and FVI : carbon steel (CS) and aluminium (Al) as the workpiece whereas, tungestin carbide (WC) as the cutting tool N.B.  high wettability is indicated by a smaller contact angle value.

Contact angle measurements for water, commercial sample, and cutting fluids formulas (Formula I, Formula VI) on the workpieces (CS and Al), and tool (WC) are shown in Fig. 16. The formulas have contact angles that are smaller than those of water and are compatible with commercial samples. That is, the formulas can completely wet the surface, implying that the formulas have better wettability. For the formulas, the contact angle varied from 25.84, 25.22 on CS, 24.90, 23.68 on Al, and 23.15, 22.28 on WC for formulas I and VI, respectively. Given that wettability typically correlates with the surface tension of a fluid, the measured surface tensions (*γ*_LG_) were found to be similar (31 and 29 for Formula I and Formula VI). Therefore, the disparity in contact angles observed between fluids is likely attributable to their varying interfacial tensions (*γ*_SL_) with solid surfaces, as the (*γ*_SG_) value remained constant in Eq. (1) ^76^. Additionally, the findings indicate that the effectiveness of the formulas in wetting WC was generally high compared to CS and Al. Finally, the test findings demonstrated that Formula VI had better wettability.

#### Droplet size and photographic studies

The droplet size of the dispersed phase in a cutting fluid nanoemulsion is an important parameter that can affect the performance of the fluid. In general, smaller droplets lead to improved cooling efficiency and lubrication, as they increase the surface area of the dispersed phase and enhance the contact between the fluid and the workpiece. Additionally, smaller droplets can reduce the tendency of the fluid to clog the cutting tool and can improve the overall stability of the fluid^77^.

**Figure 17:**
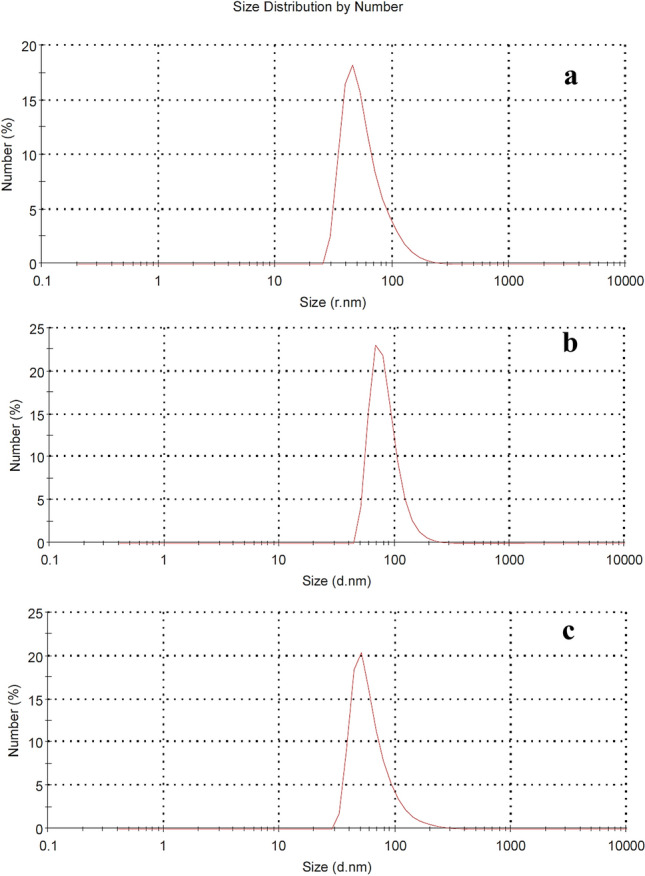
Dynamic light scattering (DLS) for (**a**) commercial sample, (**b**) Formula I, and (**c**) Formula VI.

The mean droplet size indices for the prepared emulsions, formulas I and VI, were measured at 25 °C using the DLS method for the nano-emulsions produced with different mixtures of emulsion-stabilizing surfactants. As shown in Fig. 17 (a–c), the formulas showed smaller average droplet sizes (less than 200 nm). The mean droplet sizes were 58.70, 63.97, and 83.24 nm for commercial sample, FVI, and FI, respectively. The size distribution plot for formulas exhibited uniformity. This means that the droplets are closer in size to each other leading to narrower peaks in the size distribution plot. The droplet size uniformity prevents creaming as a result of Oswald ripening. The difference between the droplet sizes of mixed blend emulsifiers can be explained through the nature and strength of the structure as well as the composition of the absorbed layer at the o/w interfaces^78^. FVI displayed smaller droplet sizes and higher droplet size uniformity than the FI. This is attributed to the negatively charged head group of anionic emulsifier in FVI which creates electrostatic repulsion impeding the close approach and coalescence of the droplet. Several studies have shown that the use of emulsifier mixtures can lead to a reduction in droplet size and an improvement in emulsion stability compared to using a single surfactant^78^.

**Figure 18:**
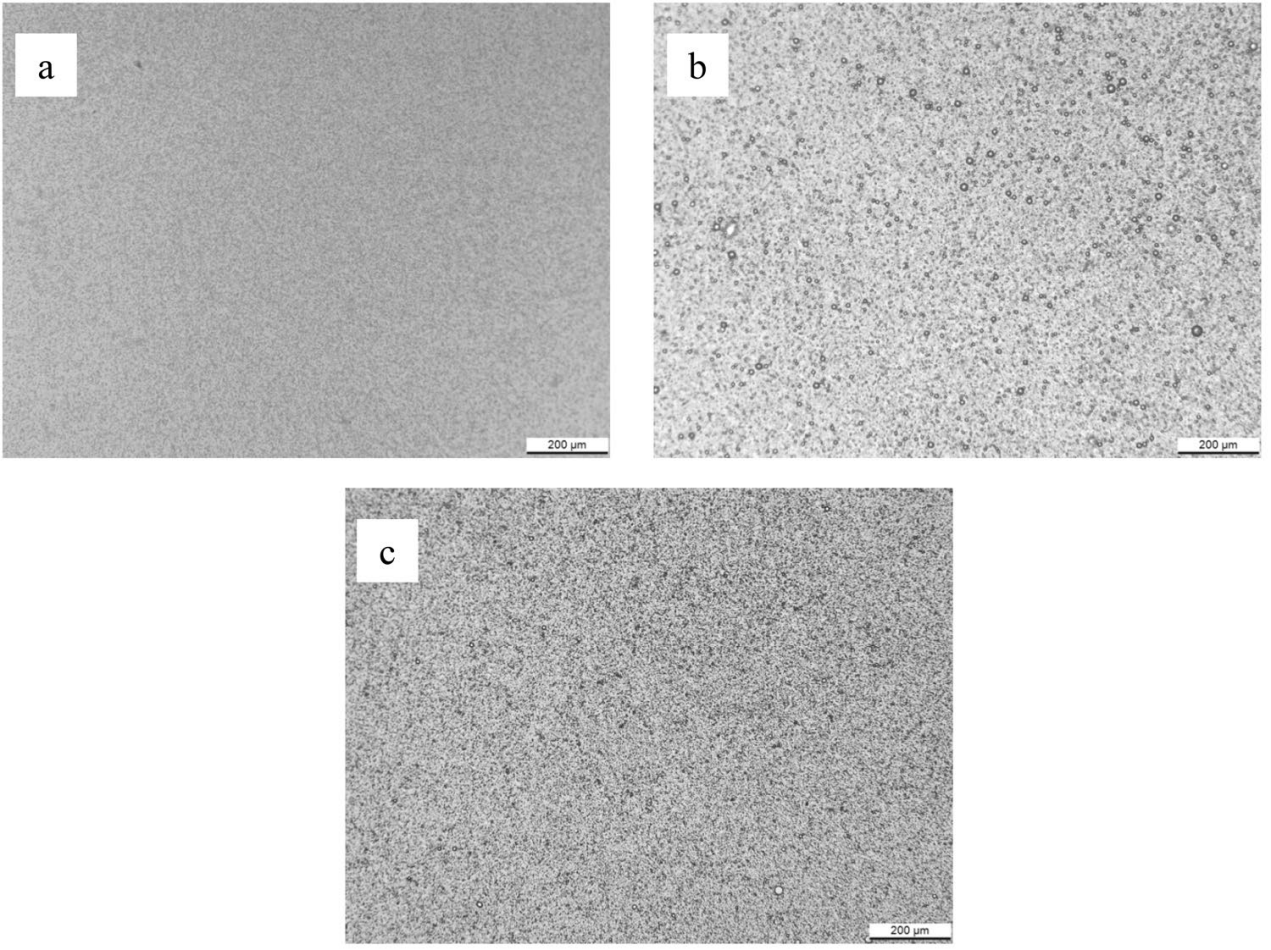
Photographic image for (**a**) commercial sample, (**b**) Formula I, and (**c**) Formula VI.

According to the photographic images in Fig. 18, the oil droplets dispersed uniformly within the continuous water phase. The mixed emulsifiers are critical in stabilizing the emulsion by producing a protective layer surrounding each oil droplet reducing droplet size and preventing coalescence. The emulsion stability is improved by the droplets' nano-size due to the droplets' consistency, which inhibits creaming^71^. Formula VI produces better outcomes in surface tension, wettability, droplet size, and photographic image testing. As a result, Formula VI was chosen to go through additional testing.

#### Tribology studies

A change in metal-cutting technology has resulted from recent tribological developments over the past few years^79^. Material and energy conservation is becoming an essential problem. Friction is the fundamental reason for energy loss in a mechanical system, but it can be reduced with lubrication. As a result, it is critical to improve the lubrication properties. The key to optimizing this procedure is a good blend of oil base and additives^80,81^.

**Figure 19:**
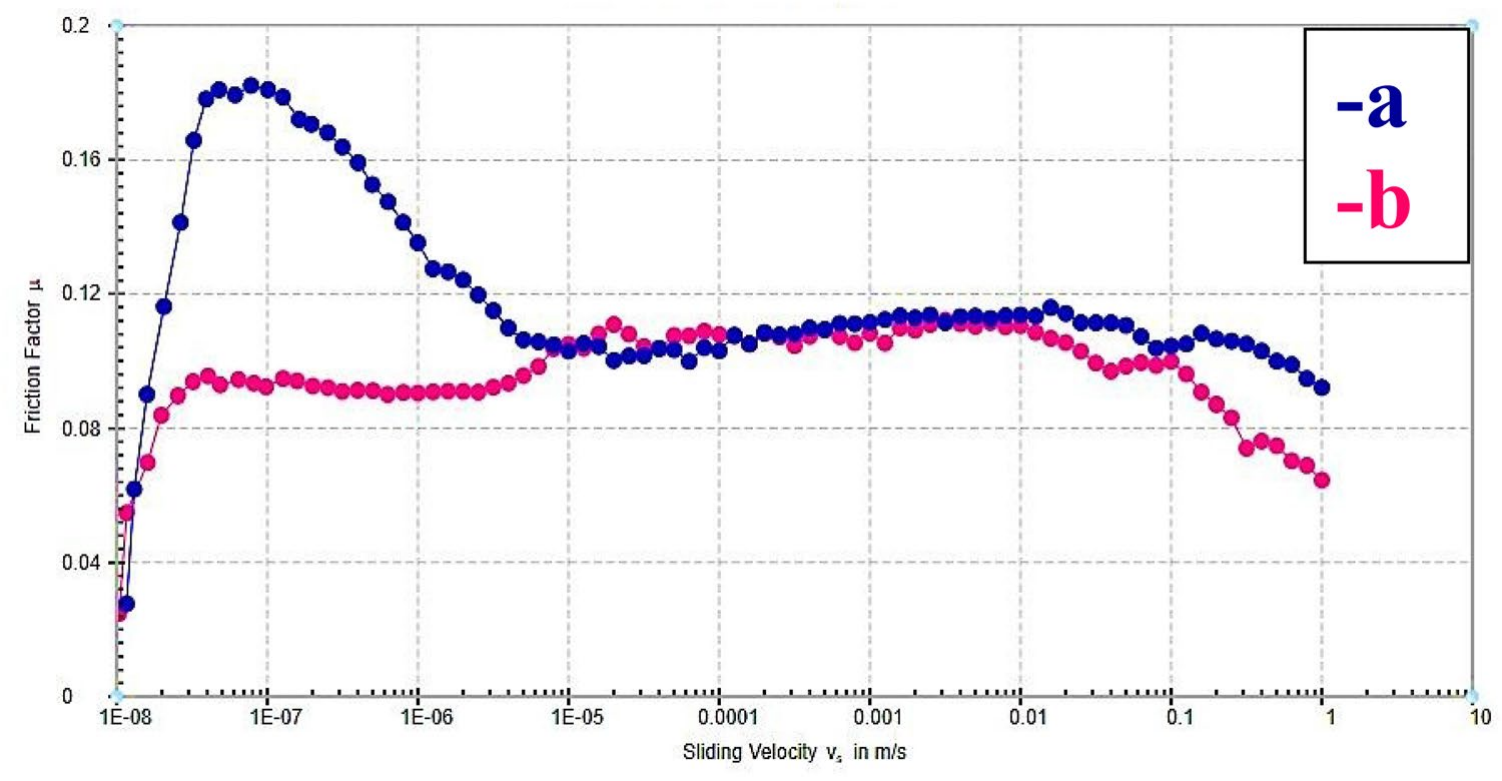
Variation of friction factor (μ) with sliding velocity (v) for; (**a**) commercial sample and (**b**) formula VI.

The friction coefficients for the commercial sample, FVI are displayed in Fig. 19 (a,b), respectively. It's interesting to note that the friction factor has a relatively large peak at short sliding velocities. This is because the film that forms to protect the metal's surface has not yet been formed. On the other hand, the friction coefficient decreases as the sliding velocity increases due to less friction between the ball and the three plates. The friction coefficient significantly decreases compared with commercial (Fig. 19a). This is due to the chemical structures of the recycled soybean oil (ester, double bonds, and long-chain alkyl groups) and mixed anionic/nonionic emulsifiers (amide, sulfate, ester, polyoxyethylene, and hydroxyl groups) that created an adsorbed film on metal surfaces, preventing metal-to-metal contact and reducing friction. Furthermore, oil's comparatively high viscosity allows it to maintain a thick and durable lubricating film, particularly under high-pressure and high-temperature conditions^82^. As the film's thickness and adhesion increase, the friction coefficient depresses^83^.

#### Cytotoxicity test

HaCaT cell lines were tailored as a model system to examine the potential toxicity of cutting fluids (commercial sample and FVI) in human keratinocyte cell lines. For FVI, MTT results showed its maximum toxicity value (32.396251673%) at 10,000 ug/ml followed by 15.662650602% at 5000 ug/ml. On the other hand, no toxicity was recorded for FVI at 2500, 1250, 625, and 312.5 ug/ml after 48 h. Furthermore, the used commercial sample caused 37.148594378% and 18.005354752% at 10,000, and 5000 ug/ml respectively, and no toxicity was recorded at 2500, 1250, 625, and 312.5 ug/ml. Recorded IC50 values were 13,520.76 and 12,570.30 ug/ml for FVI and commercial sample respectively as shown in Table 5. The cytotoxicity assay demonstrated the safety of FVI on humans and the environment. Somashekaraiah et al.^18^ evaluated the performance of two formulations, a green cutting fluid and commercial sample on HaCaT cell lines. No death was recorded on using the green at 50 ug/ml, whereas the commercial sample caused a significant cellular mortality at 50 ug/ml^18^.

**Table 5:** Investigation of cytotoxicity activities of commercial sample and FVI on (HaCaT) cell line.

ID	Conc.ug/ml	O.D			Mean O.D	ST.E	Viability %	Toxicity %	IC50
HaCaT	dilution	0.515	0.486	0.493	0.498	0.008737	100	0	
**Com**	10,000	0.301	0.320	0.318	0.313	0.006028	62.851405622	37.148594378	**12,570.30**
	5000	0.413	0.401	0.411	0.408333	0.003712	81.994645248	18.005354752	
	2500	0.501	0.498	0.492	0.497	0.002646	99.799196787	0.200803213	
	1250	0.507	0.490	0.502	0.499667	0.005044	100.334672021	− 0.334672021	
	625	0.490	0.492	0.517	0.499667	0.008686	100.334672021	− 0.334672021	
	312.5	0.495	0.497	0.509	0.500333	0.004372	100.468540830	− 0.468540830	
**FVI**	10,000	0.337	0.331	0.342	0.336667	0.00318	67.603748327	32.396251673	**13,520.76**
	5000	0.421	0.413	0.426	0.420	0.003786	84.337349398	15.662650602	
	2500	0.493	0.499	0.487	0.493	0.003464	98.995983936	1.004016064	
	1250	0.496	0.501	0.504	0.500333	0.002333	100.468540830	− 0.468540830	
	625	0.510	0.508	0.498	0.505333	0.003712	101.472556894	− 1.472556894	
	312.5	0.500	0.494	0.490	0.494667	0.002906	99.330655957	0.669344043	

Significant values are in [bold].

## Conclusion

1. The recycled vegetable oil was extracted from SBE via the cold method and its physiochemical properties were assessed.
2. The recycled vegetable oil was modified chemically to synthesize anionic and nonionic emulsifiers with various ethylene oxide units, as well as mono and gemini cationic surfactants as corrosion inhibitors and biocides.
3. According to the results, mixed emulsifiers with different ratios of nonionic/nonionic (Formulas I, II, III, and IV), and anionic/nonionic (Formula V, and VI) displayed stable emulsions when compared to individual emulsifiers.
4. Formulas I and VI showed good protective efficiency from the corrosion test.
5. The prepared eco-friendly formula VI possessed better wettability (25.22 on CS, 23.68 on Al, and 22.28 on WC) and a smaller particle size (63.97 nm).
6. Additionally, the impact of formula VI on the machine's performance was examined. According to the findings, formula VI was able to display that the friction coefficient decreases with increasing sliding velocity.
7. The cytotoxicity assay demonstrated the safety of formula VI on humans and the environment.

### Supplementary Information

Supplementary Figures.

## Data Availability

The data used and analyzed during the current study are available from the corresponding author upon reasonable request as long as the request does not compromise intellectual property interests.
