# Supplementary material for: Sustainable utilization of the vegetable oil manufacturing waste product in the formulation of eco-friendly emulsifiable cutting fluids
Source: Sci Rep. 2023 Dec 4;13:21406. doi: 10.1038/s41598-023-46768-8 (PMC10696049; doi:10.1038/s41598-023-46768-8)
Supplement: Supplementary file 1 — Supplementary Figures. [file 41598_2023_46768_MOESM1_ESM.docx]

**Supplementary Data**

**Sustainable utilization of the vegetable oil manufacturing waste product in the formulation of eco-friendly emulsifiable cutting fluids**

**Fig. (S1): Synthesis of mono and gemini cationic surfactants**

**Fig. (S2): IR spectrum of N-(2-hydroxyethyl) alkyl amide (HEA-A)**


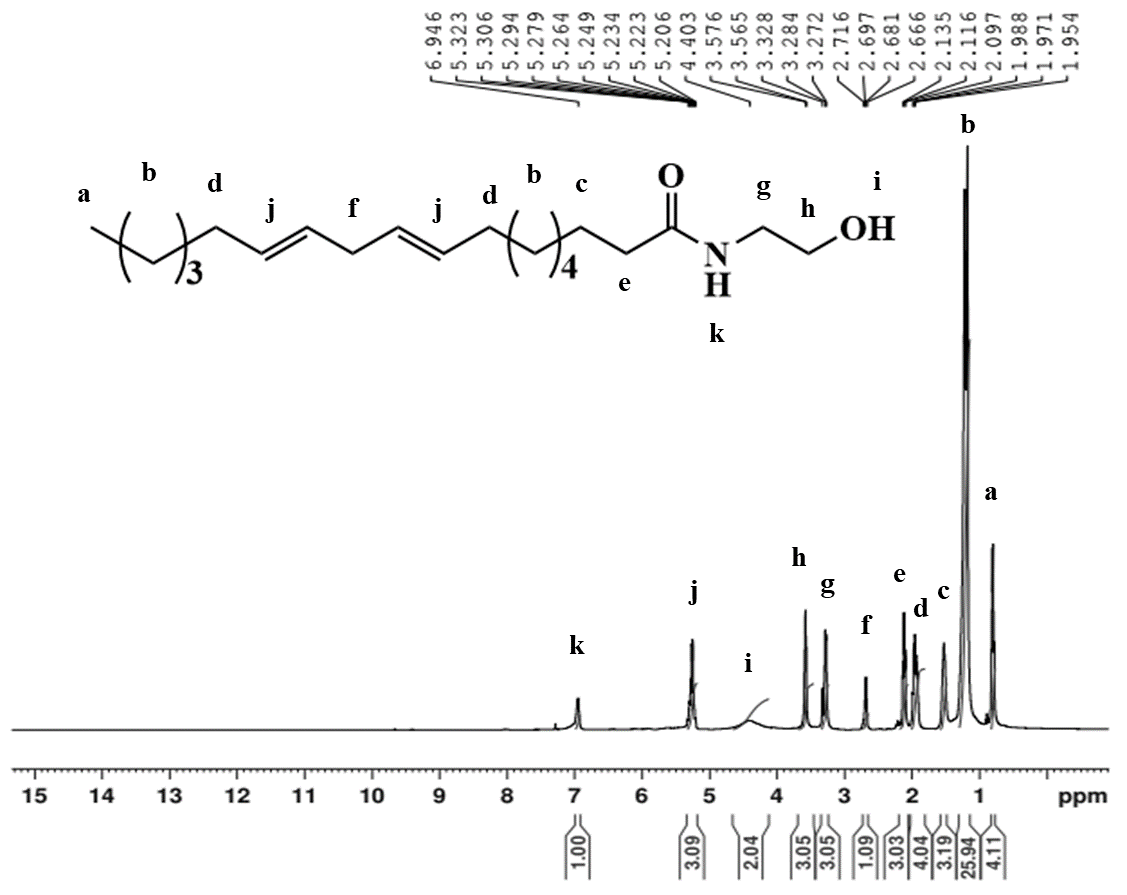


**Fig. (S3): ^1^H-NMR spectrum of N-(2-hydroxyethyl) alkyl amide (HEA-A)**

**Fig. (S4): IR spectrum of N-(2-aminoethyl) alkyl amide (AEAA)**


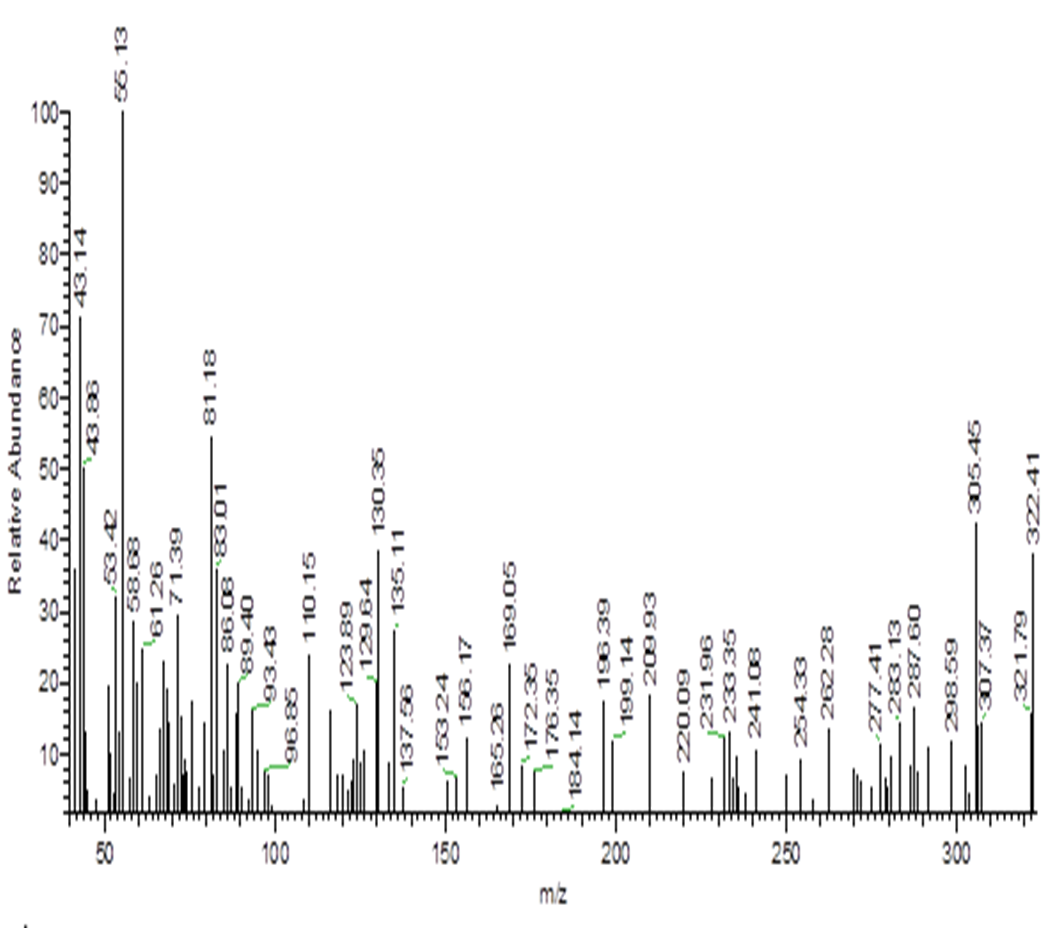


**Fig. (S5): Mass spectrum of N-(2-aminoethyl) alkyl amide (AEAA)**

**Fig. (S6): IR spectrum of 4-((2-alkylamidoethyl)amino)-4-oxobutanoic acid (AEOB)**

**Fig. (S7): IR spectrum of nonionic emulsifiers (NS)**

**Fig. (S8): IR spectrum of N-(2-alkylamidoethyl)-2-chloroacetamide (AECA)**


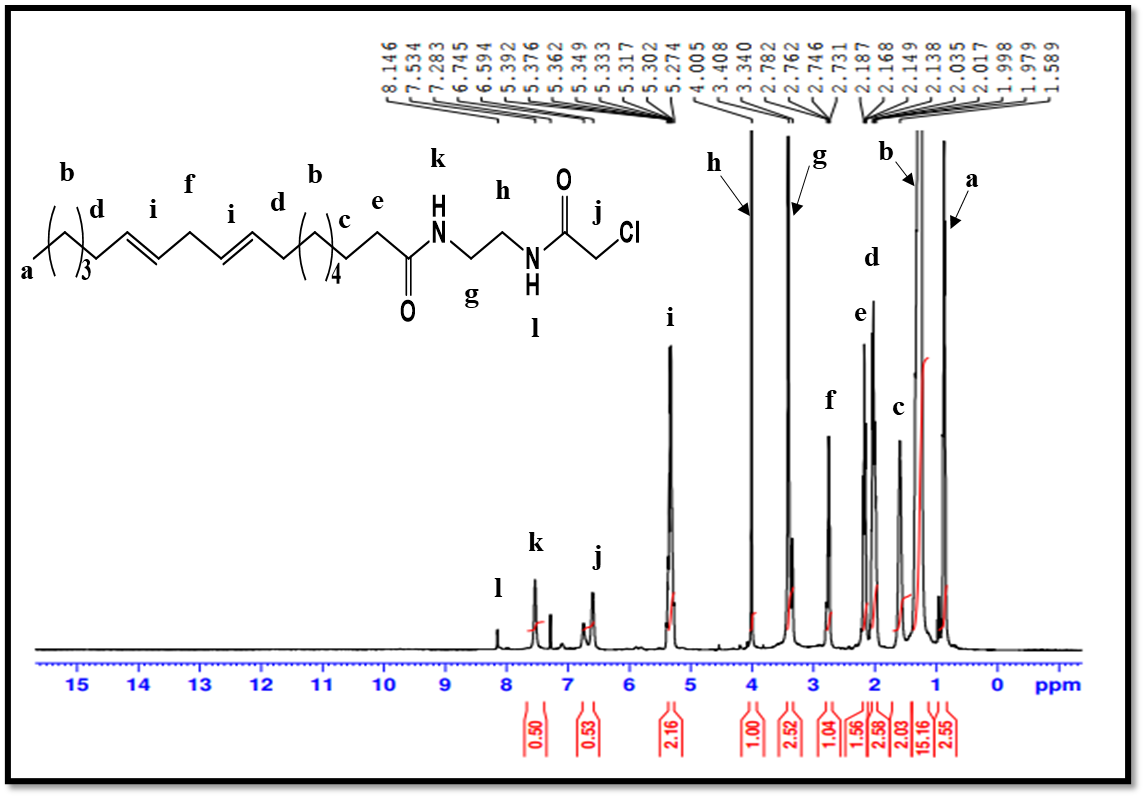


**Fig. (S9): ^1^H-NMR spectrum of N-(2-alkylamidoethyl)-2-chloroacetamide (AECA)**

**Fig. (S10): IR spectrum of 2-((2-alkylamidoethyl)amino)-N,N,N-triethyl-2-oxoethan-1-aminium chloride (MCS)**

**Fig. (S11): IR spectrum of N,N′-bis(2-((2-alkylamidoethyl)amino)-2-oxoethyl)-N,N,N′,N tetramethylethane-1,2-diaminium chloride (GCS)**
